# Risk of Cardiovascular Events and Lipid Profile Change in Patients with Breast Cancer Taking Aromatase Inhibitor: A Systematic Review and Meta-Analysis

**DOI:** 10.3390/curroncol30020142

**Published:** 2023-02-02

**Authors:** Jeong-Ju Yoo, Eun-Ae Jung, Zisun Kim, Bo-Yeon Kim

**Affiliations:** 1 Department of Internal Medicine, Soonchunhyang University Bucheon Hospital, Soonchunhyaung University College of Medicine, Bucheon 14584, Republic of Korea; 2 Department of Medical Library, Soonchunhyang University Bucheon Hospital, Soonchunhyaung University College of Medicine, Bucheon 14584, Republic of Korea; 3 Department of General Surgery, Soonchunhyang University Bucheon Hospital, Soonchunhyaung University College of Medicine, Bucheon 14584, Republic of Korea

**Keywords:** aromatase inhibitor, cardiovascular risk, angina, stroke, thromboembolism

## Abstract

Cardiovascular disease (CVD) is one of the most common comorbidities in breast cancer survivors. Recently, the target population and treatment period for aromatase inhibitor (AI) treatment in breast cancer patients has been expanding. However, information on adverse CVD events from the long-term use of AI is still lacking. The aim of this study was to investigate the CVD side effects of AI treatment and to evaluate the changes in lipid profile during AI treatment. A systematic search of PubMed (Medline), EMBASE, and Cochrane Library databases reporting on cardiovascular outcomes or lipid profiles change in adult female breast cancer patients (>19 years old) with AI was performed. The pooled analysis of 25 studies showed that the prevalence rate of any type of cardiovascular disease was 6.08 per 100 persons (95% CI 2.91–10.31). Angina was the most common type of heart-related cardiovascular event accounting for 3.85 per 100 persons, followed by any type of stroke (3.34) and venous thromboembolism (2.95). Ischemic stroke (OR 1.39, 95% CI 1.07–1.81) and myocardial infarction (OR 1.30, 95% CI 0.88–1.93) were more common in AI compared with tamoxifen, whereas the prevalence of venous thromboembolism (OR 0.61, 95% CI 0.37–1) was significantly lower in the AI group. In addition, treatment with AI for 6–12 months showed a decrease in HDL-cholesterol and an increase in LDL-cholesterol and total cholesterol. Various CVDs can occur when using AI, and in particular, the risk of MI and ischemic stroke increases in comparison with the adverse effect of tamoxifen. The occurrence of CVD might be related to the deterioration of the lipid profile after AI treatment. Therefore, a customized individualization strategy considering each patient’s CV risk factors is needed during AI treatment.

## 1. Introduction

Breast cancer is a disorder rapidly increasing in prevalence worldwide [1,2]. In the United States, breast cancer is one of the most common cancers in women after skin cancer. A woman’s lifetime chance of developing breast cancer is 12.9%, and the incidence of breast cancer is increasing at the rate of 0.5% per year [3]. Fortunately, due to the development of effective screening and various treatment methods, the mortality rate from breast cancer is decreasing every year, and the 5-year survival rate reaches more than 90% [4]. As of 2021, the number of breast cancer survivors in the United States amounted to 3.8 million. The management of these breast cancer survivors’ comorbidities is directly related to the long-term prognosis of breast cancer patients.

Cardiovascular disease (CVD) is one of the most common comorbidities in breast cancer survivors [5]. In fact, CVD and breast cancer share various risk factors, and breast cancer patients have a high prevalence of CVD compared with the general population. CVD and its risk factors should be treated well clinically, as poor management of these CVDs leads to CVD death in breast cancer survivors [6].

In 2022, the Early Breast Cancer Trialists’ Collaborative Group (EBCTCG) reported that aromatase inhibitors (AI) such as anastrozole, exemestane, or letrozole had a lower recurrence rate than tamoxifen in patients with early premenopausal hormone receptor-positive breast cancer accompanied by ovarian suppression therapy [7]. As similar evidence has been accumulated, the AI treatment period has recently been lengthened, and the indications for AI treatment in breast cancer patients have been extended to include premenopausal. However, reports of adverse events from the long-term use of AI have also been increasingly notified. Several recent studies showed that the use of AI escalates the risk of cardiac-related events and strokes in breast cancer patients [8,9,10]. These side effects of AI can be clinically problematic, especially for those breast cancer patients with high life expectancy. In addition, several studies show that the use of AI worsens the lipid profile, which is presumed to affect the increase in cardiovascular disease risk.

Despite the recent trend recommending the long-term use of AI, studies on the side effects of AI are lacking. Therefore, this study aims to investigate the CVD side effects of AI treatment in breast cancer patients through a systematic review of the literature and meta-analysis. The prevalence of CVD side effects according to AI treatment, a risk analysis of CVD outcome compared with tamoxifen, and changes in the lipid profile after AI treatment was analyzed.

## 2. Materials and Methods

The protocol for this review was pre-registered with PROSPERO (International Prospective Register of Systematic Reviews), which is an international registry for systematic reviews, and was performed according to the Preferred Reporting Items for Systematic Reviews and Meta-Analyses (PRISMA) guidelines and the Meta-analysis of Observational Studies in Epidemiology (MOOSE) checklist. The registration number is CRD42022357061.

### 2.1. Inclusion Criteria, Exclusion Criteria, and Study Outcomes

The studies included in this review were of different types, such as randomized controlled trials, cross-sectional studies, or cohort studies, which can be either prospective or retrospective in design and report on the cardiovascular outcomes of adult female breast cancer patients who were older than 19 years old and took AI as treatment. The exclusion criteria were as follows: (i) case reports, (ii) case series of fewer than five patients, and (iii) review articles. The primary outcome of the study was the prevalence of cardiovascular events after AI in patients with breast cancer. For cardiovascular outcome, the overall outcome, regardless of type, was investigated first and was then subdivided into heart, brain, or thromboembolism. The secondary outcome of the study was a change in the lipid profile after AI.

Classification by country was based on the country where the research was mainly conducted. In the case of multicenter studies, multicenter studies within one continent, such as those in Europe, were classified as European, and studies involving two or more continents were classified as ‘worldwide’.

### 2.2. Search Strategy

A search strategy was developed by searching for synonymous terms, and the keywords used in the patient/problem, intervention, comparison, and outcome (PI-CO) model can be found in the supplementary material and method section. The search was conducted on various databases such as PubMed (Medline), EMBASE, Cochrane Library, Web of Science, and KoreaMed, using medical subject headings (MeSH) and terms to identify studies that were published in English between 1 January 1990, and 31 March 2022. The search strategies and results for each database can be found in the supplementary material and method section. A professional librarian (EAJ) conducted all search processes. The search terms included index words related to breast cancer, index words related to aromatase inhibitors (AI), and index words related to cardiovascular outcomes. The lipid profile-related terms were not systematically searched as an outcome. Instead, additional information on lipid profiles was collected from articles that searched for cardiovascular outcomes.

### 2.3. Study Selection and Data Extraction

The screening process was conducted by two authors independently, who screened the titles and abstracts. Two reviewers (JJY and BYK) also independently evaluated the full-text articles for relevance. In the case of any discrepancy, it was resolved by ZSK after a discussion. Both researchers also independently assessed the risk of bias and extracted study data, including the characteristics and results, and recorded them in a standard form.

### 2.4. Methodological Quality and Risk of Bias Assessment

We used the Cochrane risk of bias tool for randomized trials [11] and the risk of bias assessment tool for non-randomized studies (RoBANS) [12] for cohort studies to assess the risk of bias; the overall results are shown in the Supplementary Material risk of bias section. Any discrepancy was resolved by two authors (JJY and BYK) after discussion. Publication bias was assessed using funnel plots (Supplementary materials). Publication bias was evaluated only when there were three or more integrated studies.

### 2.5. Statistical Analysis

We derived the pooled event rate using a random-effects model and the following method of calculation: (1) transform the event rate into a quantity (Freeman–Tukey variant of the arcsine square root transformed proportion), and (2) calculate the pooled event rate as the back-transformation of the weighted mean of the transformed event rate using the Mantel–Haenszel method and assuming the random-effect model. The comparison of event rates in the AI and tamoxifen group was calculated by a random-effects model as the mean difference for continuous variable and as a Freeman–Tukey variant of the arcsine square root transformed proportion for the binary variable. The comparative results were recorded as odds ratios with 95% confidence intervals. We analyzed the variation between studies using the I2 metric and the *p*-value from the Cochran Q test. The I2 metric, which is a ratio of the variance between studies to the total variance, can range from 0% to 100%. The statistical analysis was conducted using RevMan 5 (Cochrane Library) or the R programming language’s meta package, version 4.1.0 (R Foundation for Statistical Computing, Vienna, Austria).

## 3. Results

### 3.1. Characteristics of Included Studies

**Figure 1:**
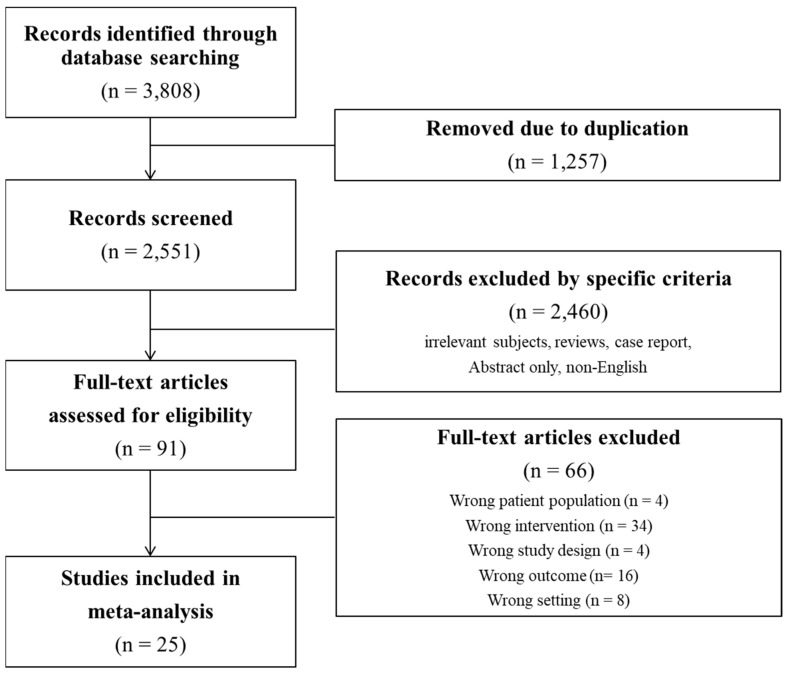
Flow charts of the study.

After reviewing the titles and abstracts, we found 91 studies that were potentially relevant. However, we eliminated 66 of these studies for specific reasons: wrong patient population (*n* = 4), wrong intervention (*n* = 34), wrong study design (*n* = 4), wrong outcome (*n* = 16), and wrong setting (*n* = 8). As a result, 25 studies were included in the meta-analysis (Figure 1). Information regarding the enrolled patients is presented in Table 1.

As shown in Table 1, all 25 studies were conducted in various countries, mostly within Europe (*n* = 10), followed by America (*n* = 8), Asia (*n* = 4), and worldwide (*n* = 3). As for the study design, the most common type was a retrospective cohort study (16 studies), followed by seven prospective RCTs and two prospective cohort studies. Of the 25 studies, 20 studies were restricted to the analysis of non-metastatic stage breast cancer, and five studies included all stages of breast cancer. Twenty-three studies were conducted on postmenopausal women, and two studies did not record menopause status, but considering the average age (67 years, 62.5 years), they were more likely to be postmenopausal women. The duration of AI drug administration was at least 3 months. A total of 22 studies compared the results of AI with other treatments (tamoxifen or placebo), and three studies analyzed only the AI-only group without a comparator.

### 3.2. AI Use and the Prevalence of Various Cardiovascular Outcomes

**Figure 2:**
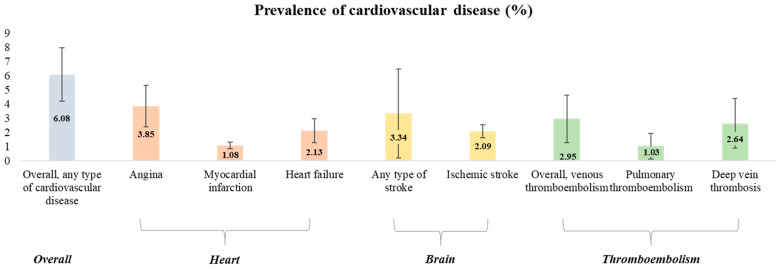
Prevalence of overall cardiovascular outcomes in patients with AI.

First, we calculated the prevalence of various cardiovascular events after AI in post-menopausal breast cancer patients (Table 2, Figure 2). The pooled prevalence rate of any type of cardiovascular disease from six studies was 6.08 per 100 persons (95% CI 2.91–10.31). Next, the cardiovascular event was sub-analyzed and divided into three categories: heart, brain, and thromboembolism. Angina was the most common type of heart-related cardiovascular event accounting for 3.85 per 100 persons (95% CI 1.48–7.18), followed by heart failure (pooled prevalence 2.13 per 100 persons, 95% CI 0.79–4.48), and myocardial infarction (pooled prevalence 1.08 per 100 persons, 95% CI 0.61–1.65). For brain-related cardiovascular events, any type of stroke and ischemic stroke were reported as 3.34 (95% CI 0–12.81) and 2.09 per 100 persons (95% CI 1.21–3.21), respectively. Finally, the overall prevalence rates of thromboembolism, pulmonary embolism, and deep vein thrombosis were 2.95 (95% CI 0.55–7.12), 1.03 (95% CI 0.01–3.61), and 2.64 per 100 persons (95% CI 0.3–7.19), respectively. The forest plots and funnel plots for each outcome are presented in Supplementary Figure S1.

### 3.3. Comparison of Various Cardiovascular Outcomes between AI and Tamoxifen

**Figure 3:**
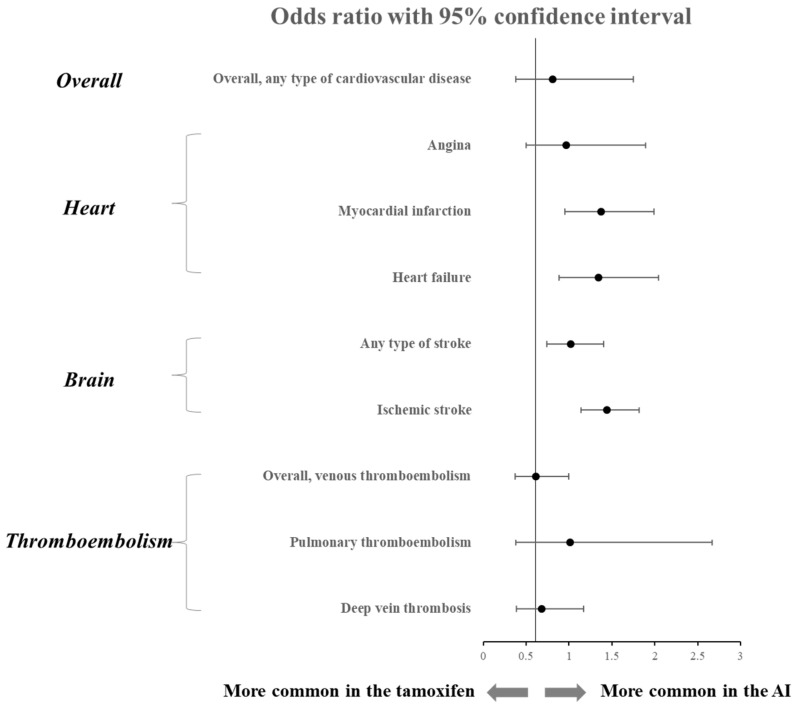
Comparison of cardiovascular outcomes between AI and tamoxifen.

For studies comparing tamoxifen and AI, we analyzed how the two drugs differed in their influence on cardiovascular outcomes (Table 3). The overall cardiovascular outcome was slightly lower than that of tamoxifen in the AI group, with a pooled odds ratio (OR) of 0.81, but it was not statistically significant (95% CI 0.38–1.75). The prevalence of the ischemic stroke was OR 1.39, which was significantly higher in the AI group than tamoxifen (95% CI 1.07–1.81). In addition, the heart-related cardiovascular events, myocardial infarction (OR 1.30, 95% CI 0.88–1.93), and heart failure (OR 1.20, 95% CI 0.78–1.86) occurred relatively more frequently in the AI group but were not statistically significant. On the other hand, the prevalence of overall thromboembolism was significantly lower in the AI group than that of the tamoxifen group with an OR of 0.61 (95% CI 0.37–1.0), and the prevalence of DVT also showed a low prevalence tendency in the AI group (OR 0.68, 95% CI 0.39–1.17). Meanwhile, the occurrence of other cardiovascular events did not show a significant difference between the AI and the tamoxifen group. Pooled forest plots and forest plots of each outcome are presented in Figure 3 and Supplementary Figure S2, respectively.

### 3.4. Change in Lipid Profile during AI Treatment

Lastly, we investigated the change in the lipid profile after AI treatment (Table 4). After the use of AI, the decrease in HDL-cholesterol was significant (pooled mean difference −2.47 mg/dL, 95% CI −4.26–−0.69) at 6 months compared with the baseline value. In addition, a tendency to increase LDL-cholesterol and total cholesterol levels were observed after the use of AI, but it was not statistically significant. When AI and tamoxifen-treated patients were compared, a significant increase in LDL-cholesterol was observed at 6 months in the AI group (pooled mean difference +6.48 mg/dL, 95% CI 0.64–16.32). The forest plots and funnel plots of each indicator are presented in Supplementary Figure S3.

## 4. Discussion

Through this meta-analysis, our study provided information about (i) the overall incidence of CVD outcomes after AI treatment, (ii) a comparison of the CVD outcome occurrence between the AI group and the tamoxifen group, and (iii) changes in the lipid profile after AI use. Because CVD is associated with the long-term mortality of patients, we assumed the importance of evaluating the CV risk even when patients had a clinical benefit to extending the duration of AI treatment in the perspective of cancer.

The first finding of our study is the incidence and types of CVD outcomes after AI treatment. Compared with the substantial meta-analysis results for the CVD outcome after tamoxifen, the research results for CVD outcome after AI are relatively insufficient [22,24,35,36]. Considering that the characteristics of the patient groups using AI or tamoxifen are different and that the two drugs are not often interchanged, the results of the AI alone group can provide a considerable amount of clinical information. To date, two meta-studies have been conducted on whether AI increases CVD in breast cancer patients [35,37]. However, the results of the two meta-analyses were inconsistent. In a meta-analysis published in 2017 [35], AI increased CVD by 19% compared to the control group, but in a meta-analysis published in 2019 [37], the increase in CVD in the AI group compared to the control group was not significant. On the other hand, in our study, certain types of CVDs, such as ischemic stroke or myocardial infarction, were analyzed to be increased in the AI group. This is probably because all meta-studies are different in terms of when the study was conducted (2017, 2019, 2022), how CVD was defined (including hypertension or dyslipidemia), and the type of study enrolled (randomized controlled trial only vs. cohort study included).

In our study, the most frequently reported CVD after AI treatment was angina, followed by any type of stroke and heart failure. However, the prevalence rates of angina, any type of stroke, and heart failure in the AI-using group were 3.85, 3.34, and 2.1 per 100 people, respectively, which was slightly lower than that of 6.2%, 20%, and 8.8% in the general women population [38,39,40]. We speculated that two factors may have contributed to the low prevalence of the AI group. First, the incidence rate might be underestimated due to the lack of interest of clinicians or the limitations of the retrospective study. Second, since the treatment period of most of the enrolled studies was less than 2 years, there is a possibility that sufficient data were not accumulated to evaluate the CVD outcome for the long-term use of AI. Nevertheless, conscientious attention from clinicians is required since it is consistently reported that AI treatment has a high CVD risk in well-designed cohort studies [8]. In particular, since the CVD outcome tends to increase with age, additional research on CVD risk in the long-term use of AI is still needed [41].

The second finding of our study was to compare the CVD prevalence between AI and tamoxifen. Although AI use had an increased risk of myocardial infarction and ischemic stroke compared with tamoxifen, there was no significant difference in heart failure or overall CVD events. Regarding the comparison of the CV risk of tamoxifen and AI, previous studies also showed inconsistent results. A population-based cohort study showed that AI slightly increased CV risk compared to tamoxifen, but it was not statistically significant as in our study [8]. On the other hand, other cohort studies reported AI to be associated more with increased risks of heart failure or cardiovascular mortality compared with tamoxifen [42]. The reasons for the inconsistency seem to be different in patient populations (ethnicity, cancer stage, prior comorbidity) or treatment durations for each study.

Meanwhile, venous thromboembolism had a higher risk in the tamoxifen-using group than in the AI-using group, which is very consistent with the previous results [43,44,45]. However, the prevalence of overall venous thromboembolism in the AI treatment group reached 2.95%, which is considerably high compared to 0.5% of the general population [46,47]. Therefore, the absolute risk of thromboembolism in the AI treatment group is never low, so this should not be overlooked in AI treatment. Heart failure was analyzed to be slightly (although not significant) higher in the AI group than tamoxifen, but it is difficult to clearly understand the mechanism by which AI treatment raises the risk of heart failure. So far, coronary artery disease is known to be the most common cause of heart failure [48]. Our results also showed a similar odds ratio between myocardial infarction and heart failure, which may provide a clue to the mechanism. Another hypothesis is that the deterioration of the lipid profile after AI treatment may exacerbate metabolic syndrome and increase the risk of heart failure as well. In fact, the risk of heart failure was 2.33 times higher in patients with metabolic syndrome consisting of high triglyceride and low HDL-cholesterol [49,50].

The third finding of our study is the change in the lipid profile after AI use. There are many related studies on how the lipid profile changes after AI use in breast cancer patients, but the results are different for each study. These differences are due to how long AI was used, in which the AI drug was used, breast cancer stage, or regularity of lipid profile data during follow-up. For example, regarding the duration of drug use, an increase in the total cholesterol and LDL-C was reported after 3 months of AI use, but there was no difference in the lipid level compared to the placebo after 36 months of use of AI [51]. In addition, among AI drugs, anastrozole and letrozole have reported lipid deterioration, but exemestane has a low rate of lipid deterioration [33,52,53]. Regarding the breast cancer stage, letrozole increased lipid levels in advanced breast cancer, but there was no significant change in the lipid before and after AI drug use in metastatic breast cancer [54,55]. Another confounding factor may be the method of collecting and recording lipid data. In the BIG 1-98 study, lipid data were collected regularly every 6 months, whereas, in the ATAC trial, data were collected irregularly on-demand [56,57].

In our study, the lipid profile after AI generally deteriorated compared to the baseline or the tamoxifen group. We thought that this change in the lipid profile might be related to the increase in CVD after AI treatment. The deterioration of the lipid profile can be explained by a phenomenon of the anti-estrogenic effect of AI. Whereas tamoxifen has an estrogenic agonistic effect on the lipid profile and CVD, AI has an anti-estrogenic effect on the lipid profile and CVD [58,59,60,61,62,63,64]. However, since there are also reports that the lipid profile deterioration of AI is comparable to tamoxifen and not significant compared to the control, well-designed follow-up studies are needed in the future. In our study, the lipid profile-related terms were not systematically searched as an outcome. Instead, additional information on the lipid profile was collected from articles that searched for cardiovascular outcomes. Therefore, the results of our meta-analysis alone are insufficient to draw accurate conclusions about the effect of AI on the lipid profile.

The strength of our study is that we analyzed well-designed studies reporting CV outcomes or lipid profile changes as primary endpoints. In particular, our meta-analysis first provided information on the lipid profile after AI. On the other hand, the limitations of our study are as follows. First, the AI treatment duration and breast cancer stage were different for each included study. Second, the majority of patients are postmenopausal women, and we cannot provide adequate information on the adverse effects of AI treatment in premenopausal women. Third, there is limited information on patients’ previous comorbidities or CV risk factors. Lastly, we could not perform in-depth investigations stratified by patients with and without lipid-lowering drugs. In these enrolled studies, detailed information on whether a statin was taken or not was disclosed to perform stratification, so integrated analysis could not be performed. This topic requires further research in the future.

## 5. Conclusions

In conclusion, various CVDs can occur when using AI, and in particular, the risk of MI and ischemic stroke increases compared with tamoxifen. On the other hand, the risk for venous thromboembolism is significantly lower in the AI group than in the tamoxifen group. The occurrence of CVD seems to be related to the deterioration of the lipid profile after AI. Therefore, customized individualization strategies considering each patient’s CV risk factors are needed when determining the duration of AI treatment. In addition, clinicians should pay attention to lipid management and educate patients on appropriate lifestyle modification when using AI.

## Figures and Tables

**Table 1:** Demographics and characteristics of studies included in the systematic review and meta-analysis.

Study	Study Type	Country	Cancer Stage	Age (Median)	Menopause	Treatment Duration	No. Group	Treatment	Control	No. Treatment	No. Control
Sawada 2005 [13]	Prospective RCT	Japan	early	58.7	postmenopausal	12 wks	2	anastrozole	TAM	22	22
Lonning 2005 [14]	Prospective RCT	Norway	early	60	postmenopausal	2 yrs	2	exemestane	placebo	58	65
Atalay 2004 [15]	Prospective RCT	Europe	non-metastatic	64	postmenopausal	48 wks	2	exemestane	TAM	36	36
Khosrow-Khavar 2020 [8]	Retrospective cohort	UK	non-metastatic	70.8	postmenopausal	>1 year	2	AI	TAM	8139	9783
Wojtacki 2001 [16]	Retrospective cohort	Poland	non-metastatic	61.6	postmenopausal	16.2 weeks	1	anastrozole		44	
Markopoulos 2009 [17]	Prospective RCT	Greece	non-metastatic	62.6	postmenopausal	5 years	2	exemestane	placebo	211	200
Tian 2018 [18]	Retrospective cohort	China	early breast cancer	59.5	postmenopausal	2 years	1	letrozole		38	
Santa-Maria 2016 [19]	Prospective cohort	USA	early breast cancer	59	postmenopausal	3 months	1	AI		422	
Abdel-Qadir 2016 [20]	Retrospective cohort	Canada	early breast cancer	71	postmenopausal	at least 1 year	2	AI	TAM	7409	1941
Pineda-Moncusi 2020 [21]	Retrospective cohort	UK, Spain	early breast cancer	67	postmenopausal	29 months	2	AI	TAM	18,455	3082
Xu 2019 [22]	Prospective cohort	USA	non-metastatic	65	postmenopausal	3.2 years	2	AI	TAM	3837	4062
Markopoulos 2005 [23]	Prospective RCT	Greece	early breast cancer	65	postmenopausal	12 months	2	exemestane	TAM	90	86
Matthews 2021 [24]	Retrospective cohort	USA, UK	early breast cancer	76	postmenopausal	2.2 years	2	AI	placebo	15,074	4667
Rabaglio 2021 [25]	Prospective RCT	Europe, USA	early breast cancer	NA	postmenopausal	5 years	2	letrozole	TAM	1535	1541
Khosrow-Khavar 2020 [9]	Retrospective cohort	Canada	early breast cancer	67.7	postmenopausal	5 years	2	AI	TAM	1962	3874
Seruga 2014 [26]	Retrospective cohort	Slovenia	early breast cancer	69	postmenopausal	NA	2	AI	TAM	33	41
Kamaraju 2019 [27]	Retrospective cohort	USA	early breast cancer	NA	postmenopausal	12 months	2	AI	TAM	4690	958
Choi 2020 [28]	Retrospective cohort	Korea	all stage	63.3	postmenopausal	3 years	2	AI	placebo	19,584	18,807
Ligibel 2012 [29]	Retrospective cohort	USA	all stage	67	NA	30 months	2	AI	placebo	9069	30,255
Faiz 2021 [30]	Retrospective cohort	USA	all stage	74.8	postmenopausal	2 years	2	AI	TAM	64,384	22,042
Franchi 2021 [31]	Retrospective cohort	Italy	early breast cancer	NA	postmenopausal	NA	2	AI	TAM	7881	7881
Chang 2022 [32]	Retrospective cohort	Taiwan	all stage	62.53	mixed	NA	2	AI	TAM	11,728	16,730
Thurlimann 2005 [33]	Prospective RCT	worldwide	early breast cancer	61	postmenopausal	25.8 months	2	letrozole	TAM	3975	3988
Sund 2021 [10]	Retrospective cohort	Sweden	early breast cancer	66	postmenopausal	46.8 months	2	AI	placebo	1481	3668
Haque 2016 [34]	Retrospective cohort	USA	all stage	66.8	postmenopausal	2.3 years	2	AI	TAM	3807	4207

Abbreviations: TAM, tamoxifen; AI, aromatase inhibitor; RCT, randomized controlled trial: NA, non-available; No., number.

**Table 2:** Prevalence of various cardiovascular outcomes in patients with breast cancer with AI.

Outcome	No. of Studies	No. of Patients Events/Total	Pooled Event Rate (per 100 Person)	95% CI	*I* ^2^	*p* for Heterogeneity
Overall, any type of cardiovascular disease	6 [21,25,31,32,33,34]	2453/47,381	6.08	2.91 to 10.31	100%	<0.01
**Heart**						
Coronary artery disease including angina	6 [21,24,25,26,33,34]	1499/42,879	3.85	1.48 to 7.18	99%	<0.01
Myocardial infarction	9 [8,9,20,24,26,28,31,32,34]	977/75,617	1.08	0.61 to 1.65	97%	<0.01
Heart failure	8 [8,9,24,25,31,32,33,34]	2338/54,101	2.13	0.79 to 4.48	100%	0
**Brain**						
Any type of stroke	2 [24,28]	1287/34,658	3.34	0 to 12.81	100%	<0.01
Ischemic stroke	5 [8,9,28,31,32]	1293/49,294	2.09	1.21 to 3.21	98%	<0.01
**Thromboembolism**						
Overall, venous thromboembolism	6 [21,22,24,25,30,33]	7503/107,260	2.95	0.55 to 7.12	100%	0
Pulmonary thromboembolism	4 [21,22,24,30]	2701/101,750	1.03	0.01 to 3.61	100%	0
Deep vein thrombosis	4 [21,22,24,30]	5866/101,750	2.64	0.30 to 7.19	100%	0

**Table 3:** Comparison of various cardiovascular outcomes between AI and tamoxifen.

Outcome	No. of Studies	No. of Patients, Events/Total (AI)	No. of Patients, Events/Total (Tamoxifen)	OR	95% CI	*I* ^2^	*p* for Heterogeneity
**Overall, any type of cardiovascular disease**	6 [21,25,31,32,33,34]	2453/47,381	3361/37,429	0.81	0.38 to 1.75	99%	<0.01
**Heart**							
Coronary artery disease including angina	6 [21,24,25,26,33,34]	1499/42,879	523/15,145	0.97	0.50 to 1.89	95%	<0.01
Myocardial infarction	9 [8,9,20,24,26,28,31,32,34]	977/75,617	412/53,824	1.30	0.88 to 1.93	88%	<0.01
Heart failure	8 [8,9,24,25,31,32,33,34]	2338/54,101	1234/50,290	1.20	0.78 to 1.86	96%	<0.01
**Brain**							
Any type of stroke	2 [24,28]	1287/34,658	237/9367	1.02	0.74 to 1.40	69%	0.07
Ischemic stroke	5 [8,9,28,31,32]	1293/49,294	833/45,349	1.39	1.07 to 1.81	85%	<0.01
**Thromboembolism**							
Overall, venous thromboembolism	6 [21,22,24,25,30,33]	7503/107,260	2893/37,001	0.61	0.37 to 1.00	97%	<0.01
Pulmonary thromboembolism	4 [21,22,24,30]	2701/101,750	991/31,472	1.01	0.38 to 2.67	94%	<0.01
Deep vein thrombosis	4 [21,22,24,30]	5866/101,750	2060/31,472	0.68	0.39 to 1.17	96%	<0.01

**Table 4:** Change in lipid profile during AI treatment.

Outcome		No. of Studies	Mean Difference	95% CI	*I* ^2^	*p* for Heterogeneity
**Comparison with baseline**						
HDL-cholesterol	6-month	5 [15,17,18,23]	−2.47	**−4.26 to −0.69**	81%	<0.01
	12-month	4 [17,18,23]	1.16	−3.18 to 5.49	94%	<0.01
LDL-cholesterol	6-month	5 [17,18,23]	10.48	−2.95 to 23.92	87%	<0.01
	12-month	5 [17,18,23]	8.05	−3.68 to 19.79	87%	<0.01
Total cholesterol	6-month	6 [15,17,18,23]	5.16	−2.07 to 12.40	83%	<0.01
	12-month	5 [17,18,23]	4.62	−4.00 to 13.24	99%	<0.01
**Comparison with tamoxifen**						
HDL-cholesterol	6-month	2 [15,23]	−3.67	−12.31 to 4.97	99%	<0.01
	12-month	2 [17,23]	−1.52	−4.79 to 7.75	99%	<0.01
LDL-cholesterol	6-month	2 [17,23]	6.48	**0.64 to 16.32**	99%	<0.01
	12-month	2 [17,23]	11.87	−15.77 to 39.50	100%	<0.01
Total cholesterol	6-month	3 [15,17,23]	1.28	−14.97 to 17.53	100%	<0.01
	12-month	2 [17,23]	6.89	−20.75 to 34.52	100%	<0.01

## Data Availability

The datasets generated during and/or analyzed during the current study are available from the corresponding author on reasonable request.

## Supplementary Materials

The following supporting information can be downloaded at: https://www.mdpi.com/article/10.3390/curroncol30020142/s1, Figure S1: Forest plots and funnel plots of Table 2; Figure S2: Forest plots and funnel plots of Table 3; Figure S3: Forest plots and funnel plots of Table 4.
