# Supplementary material for: Risk of Cardiovascular Events and Lipid Profile Change in Patients with Breast Cancer Taking Aromatase Inhibitor: A Systematic Review and Meta-Analysis
Source: Curr Oncol. 2023 Feb 2;30(2):1831–43. doi: 10.3390/curroncol30020142 (PMC9955032; doi:10.3390/curroncol30020142)
Supplement: Supplementary file 1 [file curroncol-30-00142-s001.zip › curroncol-2171824-supplementary.pdf]

Supplementary Material

Table of Contents

I. Supplementary Materials and Methods……………………………………………..……..……2

Search strategy and results

Risk of bias

II. Supplementary Figures....……………..………………………………..……..……..…13

I. Supplementary Materials and Methods

1. Search strategy and results

(1) Summary of search results

No.	DB	Results	Duplication
1	PubMed (Medline)	999	
2	EMBASE	1,717	
3	Cochrane Library	522	1,256
4	Web of Science	567	
5	KoreaMed	3	
Number of Search Results (including duplication)		3,808	
**Number of Search Results (without duplication)**		**2,552**	

(2) Keywords

PICO	Fields	Keywords	Remarks
**P**	MeSH	Breast neoplasms	**A**
	TIAB	Breast neoplasm*	**B**
	TIAB	Breast tumor*	
	TIAB	Breast carcinoma*	
	TIAB	Human mammary neoplasm*	
	TIAB	Breast cancer*	
	TIAB	Mammary neoplasm*	
	TIAB	Mammary cancer*	
	TIAB	Mammary carcinoma*	
	TIAB	Mammary tumor*	
	TIAB	Breast malignant neoplasm*	
	TIAB	Breast malignant tumor*	
	TIAB	Breast malignant cancer*	
	TIAB	Breast malignant carcinoma*	
		**A OR B**	**C**
**I**	MeSH	Tamoxifen	**D**
	MeSH	Toremifene	
	MeSH	Letrozole	
	MeSH	Anastrozole	

	MeSH	Antineoplastic agents, hormonal	
	MeSH	Selective estrogen receptor modulators	
	MeSH	Aromatase inhibitors	
	Supplementary Concept	Exemestane	**E**
	TIAB	Tamoxifen*	**F**
	TIAB	ICI-47699	
	TIAB	ICI47699	
	TIAB	Nolvadex	
	TIAB	Novaldex	
	TIAB	Tamoxifen citrate	
	TIAB	Tomaxithen	
	TIAB	Zitazonium	
	TIAB	ICI-46474	
	TIAB	ICI46474	
	TIAB	ICI-46,474	
	TIAB	ICI46,474	
	TIAB	Soltamox	
	TIAB	Crisafeno	
	TIAB	Toremifene	
	TIAB	Fareston	
	TIAB	FC-1157a	
	TIAB	FC1157a	
	TIAB	CGS-20267	
	TIAB	CGS20267	
	TIAB	Femara	
	TIAB	Letrozole	
	TIAB	Anastrazole	
	TIAB	Anastrozole	
	TIAB	ICI D1033	
	TIAB	ZD-1033	
	TIAB	ZD1033	
	TIAB	Arimidex	
	TIAB	Exemestane	
	TIAB	Examestane	
	TIAB	FCE 24304	
	TIAB	FCE24304	
	TIAB	Aromasin	
	TIAB	Hormonal antineoplast*	

	TIAB	Antineoplastic hormon*	
	TIAB	SERM*	
	TIAB	Selective estrogen receptor modulator*	
	TIAB	Aromatase inhibitor*	
		**D OR E OR F**	**G**
**O**	MeSH	Angina pectoris	**H**
	MeSH	Aortic valve stenosis	
	MeSH	Arrhythmias, cardiac	
	MeSH	Atherosclerosis	
	MeSH	Atrial fibrillation	
	MeSH	Cardiomyopathies	
	MeSH	Cardiovascular diseases	
	MeSH	Coronary artery disease	
	MeSH	Embolism	
	MeSH	Heart failure	
	MeSH	Myocardial infarction	
	MeSH	Myocardial ischemia	
	MeSH	Myocarditis	
	MeSH	Peripheral vascular diseases	
	MeSH	Stroke	
	MeSH	Thromboembolism	
	MeSH	Venous thromboembolism	
	MeSH	Heart diseases	
	TIAB	Acute stroke*	**I**
	TIAB	Angina	
	TIAB	Angor pectoris	
	TIAB	Aortic stenos*	
	TIAB	Aortic valve stenos*	
	TIAB	Apoplexy	
	TIAB	Arrhythmia	
	TIAB	Arrythmia	
	TIAB	Atherogenesis	
	TIAB	Atheroscleros*	
	TIAB	Atrial fibrillation*	
	TIAB	Auricular fibrillation*	
	TIAB	Brain vascular accident*	
	TIAB	Cardiac arrhythmia*	
	TIAB	Cardiac decompensation*	

	TIAB	Cardiac disease*	
	TIAB	Cardiac dysrhythmia*	
	TIAB	Cardiac event*	
	TIAB	Cardiac failure	
	TIAB	Cardiomyopath*	
	TIAB	Cardiovascular disease*	
	TIAB	Cardiovascular event*	
	TIAB	Cardiovascular outcome*	
	TIAB	Cardiovascular stroke*	
	TIAB	Carditis	
	TIAB	Cerebral stroke*	
	TIAB	Cerebrovascular accident*	
	TIAB	Cerebrovascular apoplexy	
	TIAB	Cerebrovascular stroke*	
	TIAB	Coronary arterioscleros*	
	TIAB	Coronary artery disease*	
	TIAB	Coronary atheroscleros*	
	TIAB	Embolism*	
	TIAB	Embolus	
	TIAB	Heart attack*	
	TIAB	Heart decompensation*	
	TIAB	Heart disease*	
	TIAB	Heart event*	
	TIAB	Heart failure	
	TIAB	Ischaemic heart disease*	
	TIAB	Ischemic heart disease*	
	TIAB	Major adverse cardiac event*	
	TIAB	Myocardial decompensation*	
	TIAB	Myocardial disease*	
	TIAB	Myocardial failure	
	TIAB	Myocardial infarct*	
	TIAB	Myocardial ischaemi*	
	TIAB	Myocardial ischemi*	
	TIAB	Myocardiopath*	
	TIAB	Myocarditides	
	TIAB	Myocarditis	
	TIAB	Paroxysmal atrial	
	TIAB	Peripheral angiopath*	

	TIAB	Peripheral vascular disease*	
	TIAB	Stenocardia*	
	TIAB	Stroke*	
	TIAB	Thromboembolism*	
	TIAB	Transient ischaemi*	
	TIAB	Transient ischemi*	
		**H OR I**	**J**
		**C AND G AND J**	**K**

(3) Search strategy

DB	Search Strategy
**PubMed**	(("breast neoplasms"[MeSH Terms] OR ("breast neoplasm*"[Title/Abstract] OR "breast tumor*"[Title/Abstract] OR "breast carcinoma*"[Title/Abstract] OR "human mammary neoplasm*"[Title/Abstract] OR "breast cancer*"[Title/Abstract] OR "mammary neoplasm*"[Title/Abstract] OR "mammary cancer*"[Title/Abstract] OR "mammary carcinoma*"[Title/Abstract] OR "mammary tumor*"[Title/Abstract] OR "breast malignant neoplasm*"[Title/Abstract] OR "breast malignant tumor*"[Title/Abstract])) AND ("tamoxifen"[MeSH Terms] OR "Toremifene"[MeSH Terms] OR "Letrozole"[MeSH Terms] OR "Anastrozole"[MeSH Terms] OR "antineoplastic agents, hormonal"[MeSH Terms] OR "selective estrogen receptor modulators"[MeSH Terms] OR "aromatase inhibitors"[MeSH Terms] OR "Exemestane"[Supplementary Concept] OR ("tamoxifen*"[Title/Abstract] OR "ICI-47699"[Title/Abstract] OR "Nolvadex"[Title/Abstract] OR "Novaldex"[Title/Abstract] OR "Tamoxifen citrate"[Title/Abstract] OR "Zitazonium"[Title/Abstract] OR "ICI-46474"[Title/Abstract] OR "ICI46474"[Title/Abstract] OR "ICI-46,474"[Title/Abstract] OR "Soltamox"[Title/Abstract] OR "Crisafeno"[Title/Abstract] OR "Toremifene"[Title/Abstract] OR "Fareston"[Title/Abstract] OR "FC-1157a"[Title/Abstract] OR "FC1157a"[Title/Abstract] OR "CGS-20267"[Title/Abstract] OR "CGS20267"[Title/Abstract] OR "Femara"[Title/Abstract] OR "Letrozole"[Title/Abstract] OR "Anastrazole"[Title/Abstract] OR "Anastrozole"[Title/Abstract] OR "ICI D1033"[Title/Abstract] OR "ZD-1033"[Title/Abstract] OR "ZD1033"[Title/Abstract] OR "Arimidex"[Title/Abstract] OR "Exemestane"[Title/Abstract] OR "Examestane"[Title/Abstract] OR "FCE 24304"[Title/Abstract] OR "FCE24304"[Title/Abstract] OR "Aromasin"[Title/Abstract] OR "hormonal antineoplast*"[Title/Abstract] OR "antineoplastic hormon*"[Title/Abstract] OR "serm*"[Title/Abstract] OR "selective estrogen receptor modulator*"[Title/Abstract] OR "aromatase inhibitor*"[Title/Abstract])) AND ("acute stroke*"[Title/Abstract] OR "Angina"[Title/Abstract] OR "Angor pectoris"[Title/Abstract] OR "aortic stenos*"[Title/Abstract] OR "aortic valve stenos*"[Title/Abstract] OR "Apoplexy"[Title/Abstract] OR "Arrhythmia"[Title/Abstract] OR "Arrythmia"[Title/Abstract] OR "Atherogenesis"[Title/Abstract] OR "atheroscleros*"[Title/Abstract] OR "atrial fibrillation*"[Title/Abstract] OR "auricular fibrillation*"[Title/Abstract] OR "brain vascular accident*"[Title/Abstract] OR "cardiac arrhythmia*"[Title/Abstract] OR "cardiac decompensation*"[Title/Abstract] OR "cardiac disease*"[Title/Abstract] OR "cardiac dysrhythmia*"[Title/Abstract] OR "cardiac event*"[Title/Abstract] OR "Cardiac failure"[Title/Abstract] OR "cardiomyopath*"[Title/Abstract] OR "cardiovascular disease*"[Title/Abstract] OR "cardiovascular event*"[Title/Abstract] OR "cardiovascular outcome*"[Title/Abstract] OR "cardiovascular stroke*"[Title/Abstract] OR "Carditis"[Title/Abstract] OR "cerebral stroke*"[Title/Abstract] OR "cerebrovascular accident*"[Title/Abstract] OR "Cerebrovascular apoplexy"[Title/Abstract] OR "cerebrovascular stroke*"[Title/Abstract] OR "coronary arterioscleros*"[Title/Abstract] OR "coronary artery disease*"[Title/Abstract] OR "coronary atheroscleros*"[Title/Abstract] OR "embolism*"[Title/Abstract] OR "Embolus"[Title/Abstract] OR "heart attack*"[Title/Abstract] OR "heart decompensation*"[Title/Abstract] OR "heart disease*"[Title/Abstract] OR "heart event*"[Title/Abstract] OR "Heart failure"[Title/Abstract] OR "ischaemic heart disease*"[Title/Abstract] OR "ischemic heart disease*"[Title/Abstract] OR "major adverse cardiac event*"[Title/Abstract] OR "myocardial decompensation*"[Title/Abstract] OR "myocardial disease*"[Title/Abstract] OR "Myocardial failure"[Title/Abstract] OR "myocardial infarct*"[Title/Abstract] OR "myocardial ischaemi*"[Title/Abstract] OR "myocardial ischemi*"[Title/Abstract] OR "myocardiopath*"[Title/Abstract] OR "Myocarditides"[Title/Abstract] OR "Myocarditis"[Title/Abstract] OR "Paroxysmal atrial"[Title/Abstract] OR "peripheral angiopath*"[Title/Abstract] OR "peripheral vascular

	disease*"[Title/Abstract] OR "stenocardia*"[Title/Abstract] OR "stroke*"[Title/Abstract] OR "thromboembolism*"[Title/Abstract] OR "transient ischaemi*"[Title/Abstract] OR "transient ischemi*"[Title/Abstract] OR ("angina pectoris"[MeSH Terms] OR "aortic valve stenosis"[MeSH Terms] OR "arrhythmias, cardiac"[MeSH Terms] OR "atherosclerosis"[MeSH Terms] OR "atrial fibrillation"[MeSH Terms] OR "cardiomyopathies"[MeSH Terms] OR "cardiovascular diseases"[MeSH Terms] OR "coronary artery disease"[MeSH Terms] OR "embolism"[MeSH Terms] OR "Heart failure"[MeSH Terms] OR "myocardial infarction"[MeSH Terms] OR ("myocardial ischemia"[MeSH Terms] OR "coronary artery disease"[MeSH Terms]) OR "Myocarditis"[MeSH Terms] OR "peripheral vascular diseases"[MeSH Terms] OR "stroke"[MeSH Terms] OR "thromboembolism"[MeSH Terms] OR "venous thromboembolism"[MeSH Terms] OR "heart diseases"[MeSH Terms]))) NOT ("animals"[MeSH Terms:noexp] NOT ("animals"[MeSH Terms:noexp] AND "humans"[MeSH Terms]))
**EMBASE**	((('breast tumor'/exp OR 'breast carcinoma'/exp OR 'breast cancer'/exp) OR ('breast neoplasm*':ab,ti OR 'breast tumor*':ab,ti OR 'breast carcinoma*':ab,ti OR 'human mammary neoplasm*':ab,ti OR 'breast cancer*':ab,ti OR 'mammary neoplasm*':ab,ti OR 'mammary cancer*':ab,ti OR 'mammary carcinoma*':ab,ti OR 'mammary tumor*':ab,ti OR 'breast malignant neoplasm*':ab,ti OR 'breast malignant tumor*':ab,ti OR 'breast malignant cancer*':ab,ti OR 'breast malignant carcinoma*':ab,ti)) AND (('tamoxifen'/exp OR 'toremifene'/exp OR 'letrozole'/exp OR 'anastrozole'/exp OR 'selective estrogen receptor modulator'/exp OR 'aromatase inhibitor'/exp OR 'exemestane'/exp OR 'tamoxifen citrate'/exp OR 'antineoplastic hormone agonists and antagonists'/exp) OR (tamoxifen*:ab,ti OR 'ici 47699':ab,ti OR ici47699:ab,ti OR nolvadex:ab,ti OR 'tamoxifen citrate':ab,ti OR 'tomaxithen':ab,ti OR 'zitazonium':ab,ti OR 'ici-46474':ab,ti OR 'ici46474':ab,ti OR 'ici-46,474':ab,ti OR 'ici46,474':ab,ti OR 'soltamox':ab,ti OR 'crisafeno':ab,ti OR 'toremifene':ab,ti OR 'fareston':ab,ti OR 'fc-1157a':ab,ti OR 'fc1157a':ab,ti OR 'cgs-20267':ab,ti OR 'cgs20267':ab,ti OR 'femara':ab,ti OR 'letrozole':ab,ti OR 'anastrazole':ab,ti OR 'anastrozole':ab,ti OR 'ici d1033':ab,ti OR 'zd-1033':ab,ti OR 'zd1033':ab,ti OR 'arimidex':ab,ti OR 'exemestane':ab,ti OR 'examestane':ab,ti OR 'fce 24304':ab,ti OR 'fce24304':ab,ti OR 'aromasin':ab,ti OR 'hormonal antineoplast*':ab,ti OR 'antineoplastic hormon*':ab,ti OR 'serm*':ab,ti OR 'selective estrogen receptor modulator*':ab,ti OR 'aromatase inhibitor*':ab,ti)) AND (('angina pectoris'/exp OR 'aortic valve stenosis'/exp OR 'heart arrhythmia'/exp OR 'atherosclerosis'/exp OR 'atrial fibrillation'/exp OR 'cardiomyopathy'/exp OR 'cardiovascular disease'/exp OR 'coronary artery disease'/exp OR 'embolism'/exp OR 'heart failure'/exp OR 'heart infarction'/exp OR 'heart muscle ischemia'/exp OR 'myocarditis'/exp OR 'peripheral vascular disease'/exp OR 'cerebrovascular accident'/exp OR 'thromboembolism'/exp OR 'venous thromboembolism'/exp OR 'heart disease'/exp OR 'aortic stenosis'/exp OR 'atherogenesis'/exp OR 'ambulatory electrocardiographic monitor'/exp OR 'carditis'/exp OR 'coronary artery atherosclerosis'/exp OR 'ischemic heart disease'/exp OR 'major adverse cardiac event'/exp OR 'myocardial disease'/exp OR 'paroxysmal atrial fibrillation'/exp OR 'transient ischemic attack'/exp) OR ('acute stroke*':ab,ti OR 'angina':ab,ti OR 'angor pectoris':ab,ti OR 'aortic stenos*':ab,ti OR 'aortic valve stenos*':ab,ti OR 'apoplexy':ab,ti OR 'arrhythmia':ab,ti OR 'arrythmia*':ab,ti OR 'atherogenesis':ab,ti OR 'atheroscleros*':ab,ti OR 'atrial fibrillation*':ab,ti OR 'auricular fibrillation*':ab,ti OR 'brain vascular accident*':ab,ti OR 'cardiac arrhythmia*':ab,ti OR 'cardiac decompensation*':ab,ti OR 'cardiac disease*':ab,ti OR 'cardiac dysrhythmia*':ab,ti OR 'cardiac event*':ab,ti OR 'cardiac failure':ab,ti OR 'cardiomyopath*':ab,ti OR 'cardiovascular disease*':ab,ti OR 'cardiovascular event*':ab,ti OR 'cardiovascular outcome*':ab,ti OR 'cardiovascular stroke*':ab,ti OR 'carditis':ab,ti OR 'cerebral stroke*':ab,ti OR 'cerebrovascular accident*':ab,ti OR 'cerebrovascular apoplexy':ab,ti OR 'cerebrovascular stroke*':ab,ti OR 'coronary arterioscleros*':ab,ti OR 'coronary artery disease*':ab,ti OR 'coronary atheroscleros*':ab,ti OR 'embolism*':ab,ti OR 'embolus':ab,ti OR 'heart arrhythmia*':ab,ti OR 'heart attack*':ab,ti OR 'heart decompensation*':ab,ti OR 'heart disease*':ab,ti OR 'heart event*':ab,ti OR 'heart failure':ab,ti OR 'heart muscle ischemi*':ab,ti OR 'ischaemic heart disease*':ab,ti OR 'ischemic heart disease*':ab,ti OR 'major adverse cardiac event*':ab,ti OR 'myocardial decompensation*':ab,ti OR 'myocardial disease*':ab,ti OR 'myocardial failure':ab,ti OR 'myocardial infarct*':ab,ti OR 'myocardial ischaemi*':ab,ti OR 'myocardial ischemi*':ab,ti OR 'myocardiopath*':ab,ti OR 'myocarditides':ab,ti OR 'myocarditis':ab,ti OR

	'paroxysmal atrial':ab,ti OR 'peripheral angiopath*':ab,ti OR 'peripheral vascular disease*':ab,ti OR 'stenocardia*':ab,ti OR 'stroke*':ab,ti OR 'thromboembolism*':ab,ti OR 'transient ischaemi*':ab,ti OR 'transient ischemi*':ab,ti))) NOT ('animal'/de NOT ('animal'/de AND 'human'/exp))
**Cochrane**	((MeSH descriptor: [Breast Neoplasms] explode all trees) OR ((Breast neoplasm*):ti,ab,kw OR (Breast tumor*):ti,ab,kw OR (Breast carcinoma*):ti,ab,kw OR (Human mammary neoplasm*):ti,ab,kw OR (Breast cancer*):ti,ab,kw OR (Mammary neoplasm*):ti,ab,kw OR (Mammary cancer*):ti,ab,kw OR (Mammary carcinoma*):ti,ab,kw OR (Mammary tumor*):ti,ab,kw OR (Breast malignant neoplasm*):ti,ab,kw OR (Breast malignant tumor*):ti,ab,kw OR (Breast malignant cancer*):ti,ab,kw OR (Breast malignant carcinoma*):ti,ab,kw)) AND ((MeSH descriptor: [Tamoxifen] explode all trees OR MeSH descriptor: [Toremifene] explode all trees OR MeSH descriptor: [Letrozole] explode all trees OR MeSH descriptor: [Anastrozole] explode all trees OR MeSH descriptor: [Antineoplastic Agents, Hormonal] explode all trees OR MeSH descriptor: [Selective Estrogen Receptor Modulators] explode all trees OR MeSH descriptor: [Aromatase Inhibitors] explode all trees) OR ((Tamoxifen*):ti,ab,kw OR (ICI-47699):ti,ab,kw OR (ICI47699):ti,ab,kw OR (Nolvadex):ti,ab,kw OR (Novaldex):ti,ab,kw OR (Tamoxifen citrate):ti,ab,kw OR (Tomaxithen):ti,ab,kw OR (Zitazonium):ti,ab,kw OR (ICI-46474):ti,ab,kw OR (ICI46474):ti,ab,kw OR (ICI-46,474):ti,ab,kw OR (ICI46,474):ti,ab,kw OR (Soltamox):ti,ab,kw OR (Crisafeno):ti,ab,kw OR (Toremifene):ti,ab,kw OR (Fareston):ti,ab,kw OR (FC-1157a):ti,ab,kw OR (FC1157a):ti,ab,kw OR (CGS-20267):ti,ab,kw OR (CGS20267):ti,ab,kw OR (Femara):ti,ab,kw OR (Letrozole):ti,ab,kw OR (Anastrazole):ti,ab,kw OR (Anastrozole):ti,ab,kw OR (ICI D1033):ti,ab,kw OR (ZD-1033):ti,ab,kw OR (ZD1033):ti,ab,kw OR (Arimidex):ti,ab,kw OR (Exemestane):ti,ab,kw OR (Examestane):ti,ab,kw OR (FCE 24304):ti,ab,kw OR (FCE24304):ti,ab,kw OR (Aromasin):ti,ab,kw OR (Hormonal antineoplast*):ti,ab,kw OR (Antineoplastic hormon*):ti,ab,kw OR (SERM*):ti,ab,kw OR (Selective estrogen receptor modulator*):ti,ab,kw OR (Aromatase inhibitor*):ti,ab,kw)) AND ((MeSH descriptor: [Angina Pectoris] explode all trees OR MeSH descriptor: [Aortic Valve Stenosis] explode all trees OR MeSH descriptor: [Arrhythmias, Cardiac] explode all trees OR MeSH descriptor: [Atherosclerosis] explode all trees OR MeSH descriptor: [Atrial Fibrillation] explode all trees OR MeSH descriptor: [Cardiomyopathies] explode all trees OR MeSH descriptor: [Cardiovascular Diseases] explode all trees OR MeSH descriptor: [Coronary Artery Disease] explode all trees OR MeSH descriptor: [Embolism] explode all trees OR MeSH descriptor: [Heart Failure] explode all trees OR MeSH descriptor: [Myocardial Infarction] explode all trees OR MeSH descriptor: [Myocardial Ischemia] explode all trees OR MeSH descriptor: [Myocarditis] explode all trees OR MeSH descriptor: [Peripheral Vascular Diseases] explode all trees OR MeSH descriptor: [Stroke] explode all trees OR MeSH descriptor: [Thromboembolism] explode all trees OR MeSH descriptor: [Venous Thromboembolism] explode all trees OR MeSH descriptor: [Heart Diseases] explode all trees) OR ((Acute stroke*):ti,ab,kw OR (Angina):ti,ab,kw OR (Angor pectoris):ti,ab,kw OR (Aortic stenos*):ti,ab,kw OR (Aortic valve stenos*):ti,ab,kw OR (Apoplexy):ti,ab,kw OR (Arrhythmia):ti,ab,kw OR (Arrythmia):ti,ab,kw OR (Atherogenesis):ti,ab,kw OR (Atheroscleros*):ti,ab,kw OR (Atrial fibrillation*):ti,ab,kw OR (Auricular fibrillation*):ti,ab,kw OR (Brain vascular accident*):ti,ab,kw OR (Cardiac arrhythmia*):ti,ab,kw OR (Cardiac decompensation*):ti,ab,kw OR (Cardiac disease*):ti,ab,kw OR (Cardiac dysrhythmia*):ti,ab,kw OR (Cardiac event*):ti,ab,kw OR (Cardiac failure):ti,ab,kw OR (Cardiomyopath*):ti,ab,kw OR (Cardiovascular disease*):ti,ab,kw OR (Cardiovascular event*):ti,ab,kw OR (Cardiovascular outcome*):ti,ab,kw OR (Cardiovascular stroke*):ti,ab,kw OR (Carditis):ti,ab,kw OR (Cerebral stroke*):ti,ab,kw OR (Cerebrovascular accident*):ti,ab,kw OR (Cerebrovascular apoplexy):ti,ab,kw OR (Cerebrovascular stroke*):ti,ab,kw OR (Coronary arterioscleros*):ti,ab,kw OR (Coronary artery disease*):ti,ab,kw OR (Coronary atheroscleros*):ti,ab,kw OR (Embolism*):ti,ab,kw OR (Embolus):ti,ab,kw OR (Heart attack*):ti,ab,kw OR (Heart decompensation*):ti,ab,kw OR (Heart disease*):ti,ab,kw OR (Heart event*):ti,ab,kw OR (Heart failure):ti,ab,kw OR (Ischaemic heart disease*):ti,ab,kw OR (Ischemic heart disease*):ti,ab,kw OR (Major adverse cardiac event*):ti,ab,kw OR (Myocardial decompensation*):ti,ab,kw OR (Myocardial disease*):ti,ab,kw OR (Myocardial failure):ti,ab,kw OR (Myocardial infarct*):ti,ab,kw OR (Myocardial ischaemi*):ti,ab,kw OR (Myocardial ischemi*):ti,ab,kw OR (Myocardiopath*):ti,ab,kw OR (Myocarditides):ti,ab,kw OR (Myocarditis):ti,ab,kw OR (Paroxysmal

	atrial):ti,ab,kw OR (Peripheral angiopath*):ti,ab,kw OR (Peripheral vascular disease*):ti,ab,kw OR (Stenocardia*):ti,ab,kw OR (Stroke*):ti,ab,kw OR (Thromboembolism*):ti,ab,kw OR (Transient ischaemi*):ti,ab,kw OR (Transient ischemi*):ti,ab,kw)) NOT (MeSH descriptor: [Animals] this term only NOT (MeSH descriptor: [Animals] this term only AND MeSH descriptor: [Humans] this term only))
**Web of Science**	(TI=("Breast neoplasm*" OR "Breast tumor*" OR "Breast carcinoma*" OR "Human mammary neoplasm*"OR "Breast cancer*" OR "Mammary neoplasm*" OR "Mammary cancer*" OR "Mammary carcinoma*" OR "Mammary tumor*" OR "Breast malignant neoplasm*" OR "Breast malignant tumor*" OR "Breast malignant cancer*" OR "Breast malignant carcinoma*")) OR AB=("Breast neoplasm*" OR "Breast tumor*" OR "Breast carcinoma*" OR "Human mammary neoplasm*"OR "Breast cancer*" OR "Mammary neoplasm*" OR "Mammary cancer*" OR "Mammary carcinoma*" OR "Mammary tumor*" OR "Breast malignant neoplasm*" OR "Breast malignant tumor*" OR "Breast malignant cancer*" OR "Breast malignant carcinoma*") AND (TI=("Tamoxifen*" OR "ICI-47699" OR "ICI47699" OR "Nolvadex" OR "Novaldex" OR "Tamoxifen citrate" OR "Tomaxithen" OR "Zitazonium" OR "ICI-46474" OR "ICI46474" OR "ICI-46,474" OR "ICI46,474" OR "Soltamox" OR "Crisafeno" OR "Toremifene" OR "Fareston" OR "FC-1157a" OR "FC1157a" OR "CGS-20267" OR "CGS20267" OR "Femara" OR "Letrozole" OR "Anastrazole" OR "Anastrozole" OR "ICI D1033" OR "ZD-1033" OR "ZD1033" OR "Arimidex" OR "Exemestane" OR "Examestane" OR "FCE 24304" OR "FCE24304" OR "Aromasin" OR "Hormonal antineoplast*" OR "Antineoplastic hormon*" OR "SERM*" OR "Selective estrogen receptor modulator*" OR "Aromatase inhibitor*")) OR AB=("Tamoxifen*" OR "ICI-47699" OR "ICI47699" OR "Nolvadex" OR "Novaldex" OR "Tamoxifen citrate" OR "Tomaxithen" OR "Zitazonium" OR "ICI-46474" OR "ICI46474" OR "ICI-46,474" OR "ICI46,474" OR "Soltamox" OR "Crisafeno" OR "Toremifene" OR "Fareston" OR "FC-1157a" OR "FC1157a" OR "CGS-20267" OR "CGS20267" OR "Femara" OR "Letrozole" OR "Anastrazole" OR "Anastrozole" OR "ICI D1033" OR "ZD-1033" OR "ZD1033" OR "Arimidex" OR "Exemestane" OR "Examestane" OR "FCE 24304" OR "FCE24304" OR "Aromasin" OR "Hormonal antineoplast*" OR "Antineoplastic hormon*" OR "SERM*" OR "Selective estrogen receptor modulator*" OR "Aromatase inhibitor*") AND (TI=("Acute stroke*" OR "Angina" OR "Angor pectoris" OR "Aortic stenos*" OR "Aortic valve stenos*" OR "Apoplexy" OR "Arrhythmia" OR "Arrythmia" OR "Atherogenesis" OR "Atheroscleros*" OR "Atrial fibrillation*" OR "Auricular fibrillation*" OR "Brain vascular accident*" OR "Cardiac arrhythmia*" OR "Cardiac decompensation*" OR "Cardiac disease*" OR "Cardiac dysrhythmia*" OR "Cardiac event*" OR "Cardiac failure" OR "Cardiomyopath*" OR "Cardiovascular disease*" OR "Cardiovascular event*" OR "Cardiovascular outcome*" OR "Cardiovascular stroke*" OR "Carditis" OR "Cerebral stroke*" OR "Cerebrovascular accident*" OR "Cerebrovascular apoplexy" OR "Cerebrovascular stroke*" OR "Coronary arterioscleros*" OR "Coronary artery disease*" OR "Coronary atheroscleros*" OR "Embolism*" OR "Embolus" OR "Heart attack*" OR "Heart decompensation*" OR "Heart disease*" OR "Heart event*" OR "Heart failure" OR "Ischaemic heart disease*" OR "Ischemic heart disease*" OR "Major adverse cardiac event*" OR "Myocardial decompensation*" OR "Myocardial disease*" OR "Myocardial failure" OR "Myocardial infarct*" OR "Myocardial ischaemi*" OR "Myocardial ischemi*" OR "Myocardiopath*" OR "Myocarditides" OR "Myocarditis" OR "Paroxysmal atrial" OR "Peripheral angiopath*" OR "Peripheral vascular disease*" OR "Stenocardia*" OR "Stroke*" OR "Thromboembolism*" OR "Transient ischaemi*" OR "Transient ischemi*")) OR AB=("Acute stroke*" OR "Angina" OR "Angor pectoris" OR "Aortic stenos*" OR "Aortic valve stenos*" OR "Apoplexy" OR "Arrhythmia" OR "Arrythmia" OR "Atherogenesis" OR "Atheroscleros*" OR "Atrial fibrillation*" OR "Auricular fibrillation*" OR "Brain vascular accident*" OR "Cardiac arrhythmia*" OR "Cardiac decompensation*" OR "Cardiac disease*" OR "Cardiac dysrhythmia*" OR "Cardiac event*" OR "Cardiac failure" OR "Cardiomyopath*" OR "Cardiovascular disease*" OR "Cardiovascular event*" OR "Cardiovascular outcome*" OR "Cardiovascular stroke*" OR "Carditis" OR "Cerebral stroke*" OR "Cerebrovascular accident*" OR "Cerebrovascular apoplexy" OR "Cerebrovascular stroke*" OR "Coronary arterioscleros*" OR "Coronary artery disease*" OR "Coronary atheroscleros*" OR "Embolism*" OR "Embolus" OR "Heart attack*" OR "Heart decompensation*" OR "Heart disease*" OR "Heart event*" OR "Heart failure" OR "Ischaemic heart disease*" OR "Ischemic heart disease*" OR "Major adverse cardiac event*" OR "Myocardial decompensation*" OR "Myocardial disease*" OR "Myocardial failure" OR "Myocardial infarct*" OR "Myocardial ischaemi*" OR

	“Myocardial ischemi*” OR “Myocardiopath*” OR “Myocarditides” OR “Myocarditis” OR “Paroxysmal atrial” OR “Peripheral angiopath*” OR “Peripheral vascular disease*” OR “Stenocardia*” OR “Stroke*” OR “Thromboembolism*” OR “Transient ischaemi*” OR “Transient ischemi*”)
**KoreaMed**	(("Breast neoplasms"[MH]) OR ((((((((((((("Breast neoplasm"[TIAB])) OR ("Breast tumor"[TIAB])) OR ("Breast carcinoma"[TIAB])) OR ("Human mammary neoplasm"[TIAB])) OR ("Breast cancer"[TIAB])) OR ("Mammary neoplasm"[TIAB])) OR ("Mammary cancer"[TIAB])) OR ("Mammary carcinoma"[TIAB])) OR ("Mammary tumor"[TIAB])) OR ("Breast malignant neoplasm"[TIAB])) OR ("Breast malignant tumor"[TIAB])) OR ("Breast malignant cancer"[TIAB])) OR ("Breast malignant carcinoma"[TIAB])) AND (((((((("Tamoxifen"[MH])) OR ("Toremifene"[MH])) OR ("Letrozole"[MH])) OR ("Anastrozole"[MH])) OR ("Antineoplastic agents, hormonal"[MH])) OR ("Selective estrogen receptor modulators"[MH])) OR ("Aromatase inhibitors"[MH]) OR (((((((((((((((((((((((((((((((((((((("Tamoxifen"[TIAB])) OR ("ICI-47699"[TIAB])) OR ("ICI47699"[TIAB])) OR ("Nolvadex"[TIAB])) OR ("Novaldex"[TIAB])) OR ("Tamoxifen citrate"[TIAB])) OR ("Tomaxithen"[TIAB])) OR ("Zitazonium"[TIAB])) OR ("ICI-46474"[TIAB])) OR ("ICI46474"[TIAB])) OR ("ICI-46,474"[TIAB])) OR ("ICI46,474"[TIAB])) OR ("Soltamox"[TIAB])) OR ("Crisafeno"[TIAB])) OR ("Toremifene"[TIAB])) AND ("Fareston"[TIAB])) AND ("FC-1157a"[TIAB])) OR ("FC1157a"[TIAB])) OR ("CGS-20267"[TIAB])) OR ("CGS20267"[TIAB])) OR ("Femara"[TIAB])) OR ("Letrozole"[TIAB])) OR ("Anastrazole"[TIAB])) OR ("Anastrozole"[TIAB])) OR ("ICI D1033"[TIAB])) OR ("ZD-1033"[TIAB])) OR ("ZD1033"[TIAB])) OR ("Arimidex"[TIAB])) OR ("Exemestane"[TIAB])) OR ("Examestane"[TIAB])) OR ("FCE 24304"[TIAB])) OR ("FCE24304"[TIAB])) OR ("Aromasin"[TIAB])) OR ("Hormonal antineoplast*"[TIAB])) OR ("Antineoplastic hormon*"[TIAB])) OR ("SERM*"[TIAB])) OR ("Selective estrogen receptor modulator*"[TIAB])) OR ("Aromatase inhibitor*"[TIAB])) AND ((((((((((((((((((Angina pectoris[MH])) OR (Aortic valve stenosis[MH])) OR (Arrhythmias, cardiac[MH])) OR (Atherosclerosis[MH])) OR (Atrial fibrillation[MH])) OR (Cardiomyopathies[MH])) OR (Cardiovascular diseases[MH])) OR (Coronary artery disease[MH])) OR (Embolism[MH])) OR (Heart failure[MH])) OR (Myocardial infarction[MH])) OR (Myocardial ischemia[MH])) OR (Myocarditis[MH])) OR (Peripheral vascular diseases[MH])) OR (Stroke[MH])) OR (Thromboembolism[MH])) OR (Venous thromboembolism[MH])) OR (Heart diseases[MH]) OR (("Acute stroke*"[TIAB])) OR ("Angina"[TIAB])) OR ("Angor pectoris"[TIAB])) OR ("Aortic stenos*"[TIAB])) OR ("Aortic valve stenos*"[TIAB])) OR ("Apoplexy"[TIAB])) OR ("Arrhythmia"[TIAB])) OR ("Arrythmia"[TIAB])) OR ("Atherogenesis"[TIAB])) OR ("Atheroscleros*"[TIAB])) OR ("Atrial fibrillation*"[TIAB])) OR ("Auricular fibrillation*"[TIAB])) OR ("Brain vascular accident*"[TIAB])) OR ("Cardiac arrhythmia*"[TIAB])) OR ("Cardiac decompensation*"[TIAB])) OR ("Cardiac disease*"[TIAB])) OR ("Cardiac dysrhythmia*"[TIAB])) OR ("Cardiac event*"[TIAB])) OR ("Cardiac failure"[TIAB])) OR ("Cardiomyopath*"[TIAB])) OR ("Cardiovascular disease*"[TIAB])) OR ("Cardiovascular event*"[TIAB])) OR ("Cardiovascular outcome*"[TIAB])) OR ("Cardiovascular stroke*"[TIAB])) OR ("Carditis"[TIAB])) OR ("Cerebral stroke*"[TIAB])) OR ("Cerebrovascular accident*"[TIAB])) OR ("Cerebrovascular apoplexy"[TIAB])) OR ("Cerebrovascular stroke*"[TIAB])) OR ("Coronary arterioscleros*"[TIAB])) OR ("Coronary artery disease*"[TIAB])) OR ("Coronary atheroscleros*"[TIAB])) OR ("Embolism*"[TIAB])) OR ("Embolus"[TIAB])) OR ("Heart attack*"[TIAB])) OR ("Heart decompensation*"[TIAB])) OR ("Heart disease*"[TIAB])) OR ("Heart event*"[TIAB])) OR ("Heart failure"[TIAB])) OR ("Ischaemic heart disease*"[TIAB])) OR ("Ischemic heart disease*"[TIAB])) OR ("Major adverse cardiac event*"[TIAB])) OR ("Myocardial decompensation*"[TIAB])) OR ("Myocardial disease*"[TIAB])) OR ("Myocardial failure"[TIAB])) OR ("Myocardial infarct*"[TIAB])) OR ("Myocardial ischaemi*"[TIAB])) OR ("Myocardial ischemi*"[TIAB])) OR ("Myocardiopath*"[TIAB])) OR ("Myocarditides"[TIAB])) OR ("Myocarditis"[TIAB])) OR ("Paroxysmal atrial"[TIAB])) OR ("Peripheral angiopath*"[TIAB])) OR ("Peripheral vascular disease*"[TIAB])) OR ("Stenocardia*"[TIAB])) OR ("Stroke*"[TIAB])) OR ("Thromboembolism*"[TIAB])) OR ("Transient ischaemi*"[TIAB])) OR ("Transient ischemi*"[TIAB])) Limitation Human

2. Risk of bias

1) Randomized controlled study

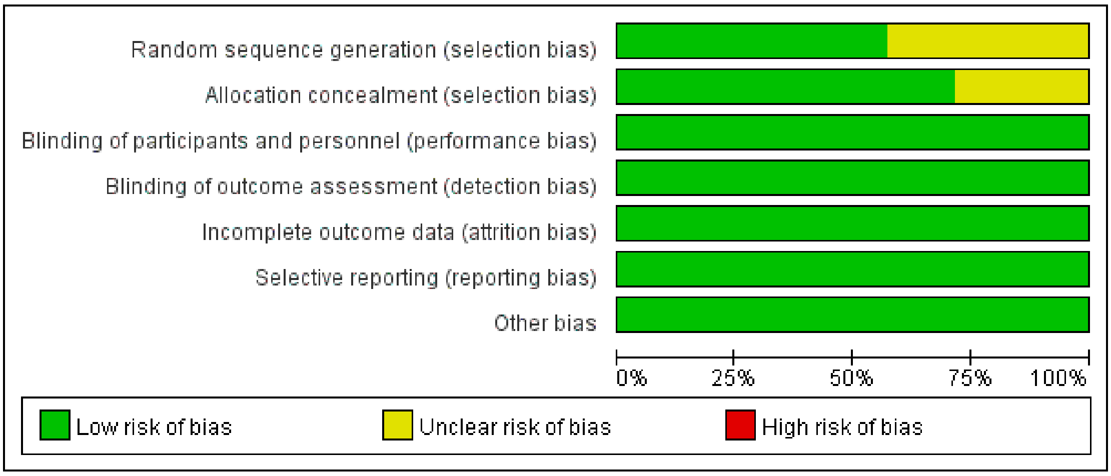

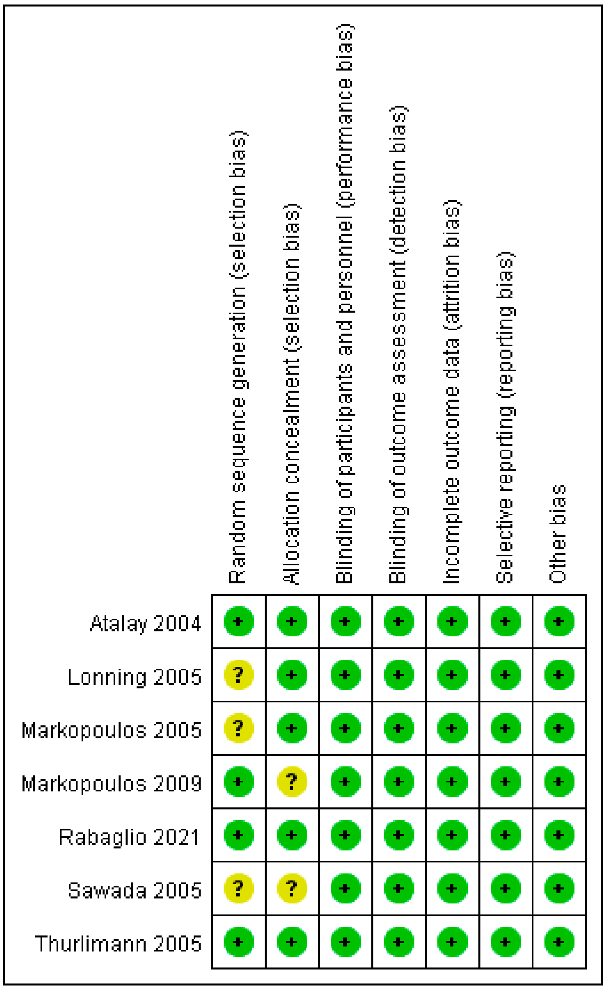

2) Non-randomized controlled study

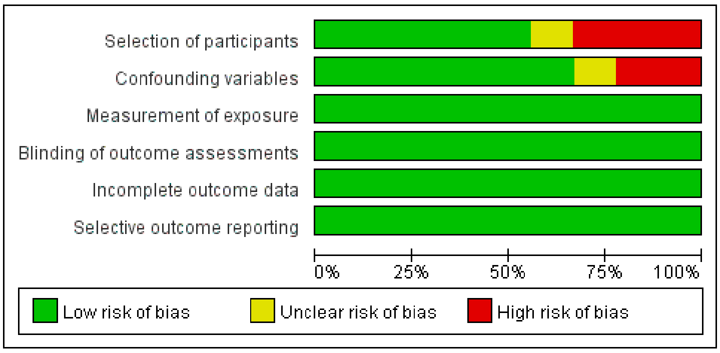

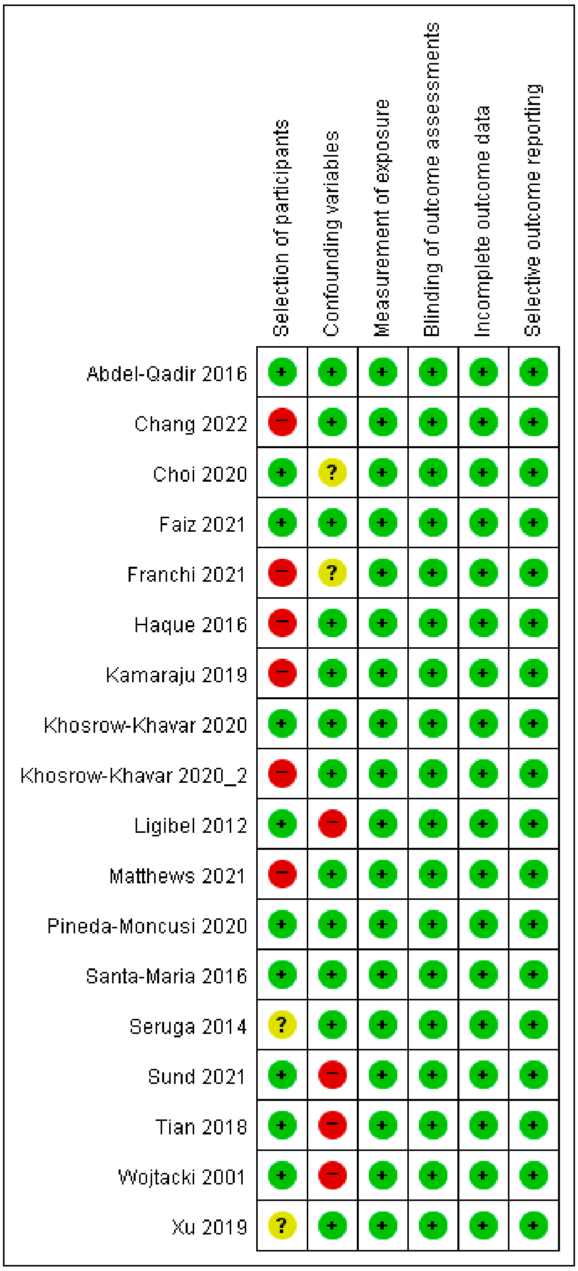

II. Supplementary Figures

Supplementary Figure S1. Forest plots and funnel plots of Table 2

<table>
<tr><td></td><td>Forest plots</td><td>Funnel Plots</td></tr>
<tr><td>Overall</td><td>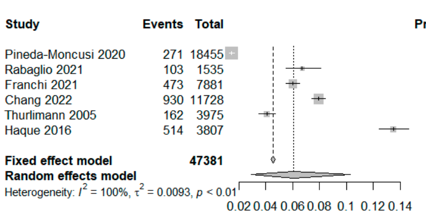
</td><td>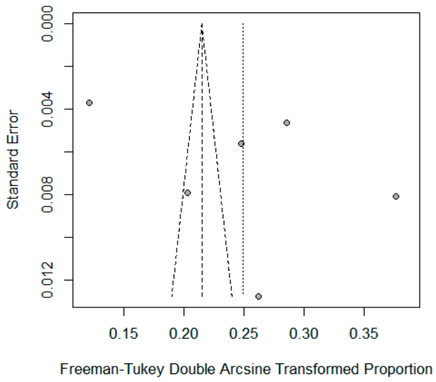
</td></tr>
<tr><td>Angina</td><td>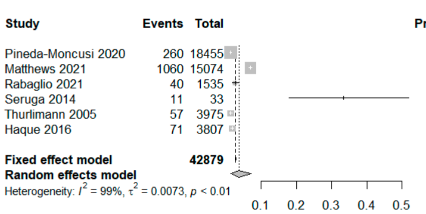
</td><td>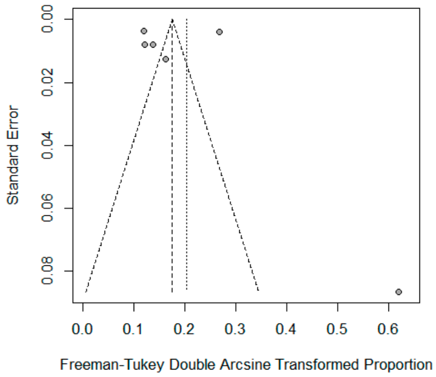
</td></tr>
<tr><td>MI</td><td>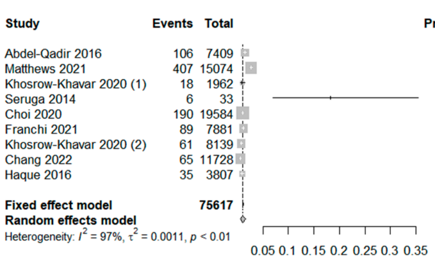
</td><td>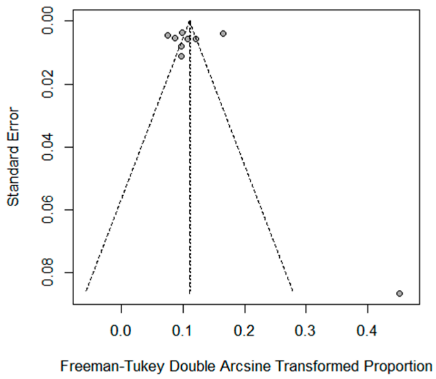
</td></tr>
</table>

Heart failure

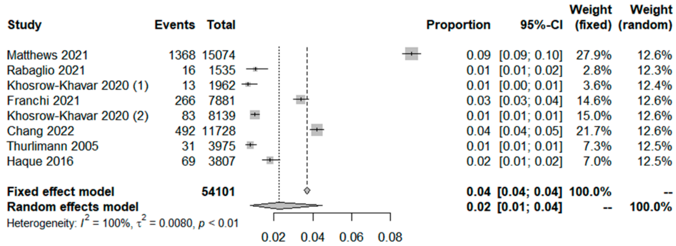

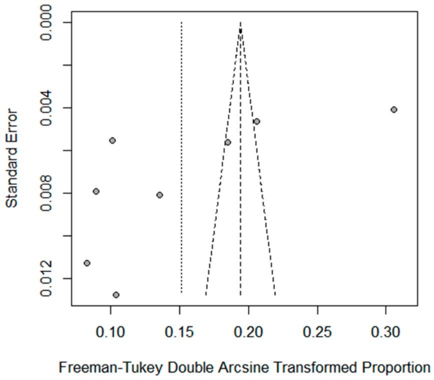

All stroke

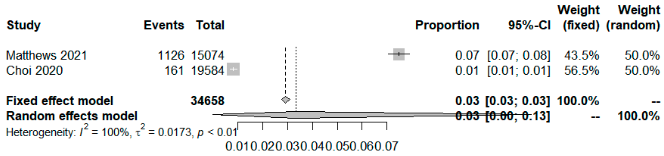

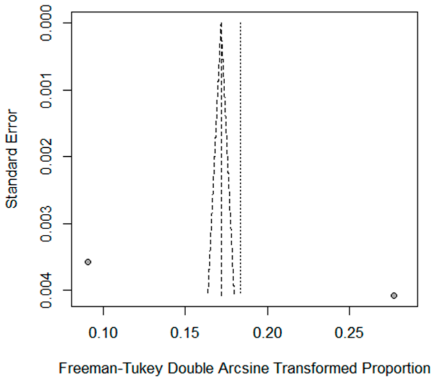

Ischemic stroke

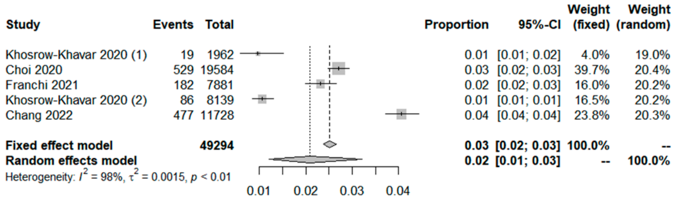

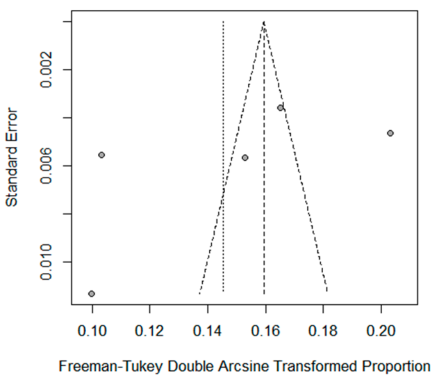

Overall, VTE

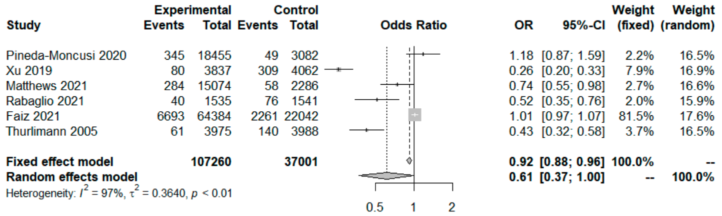

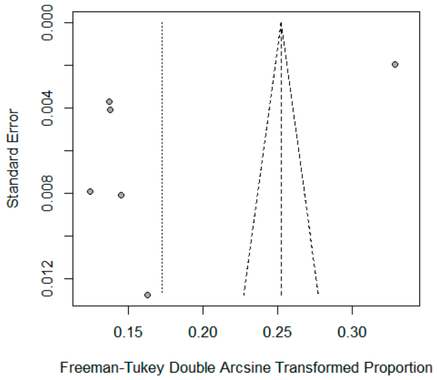

PTE

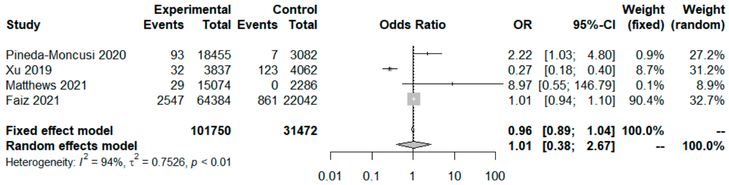

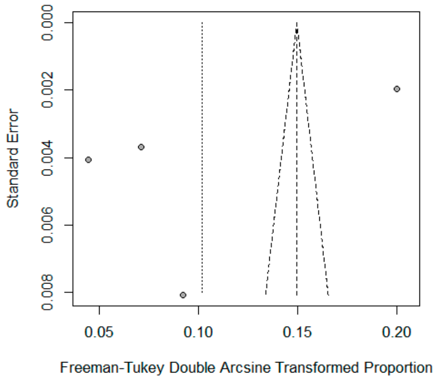

DVT

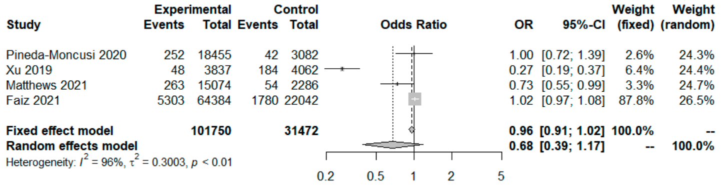

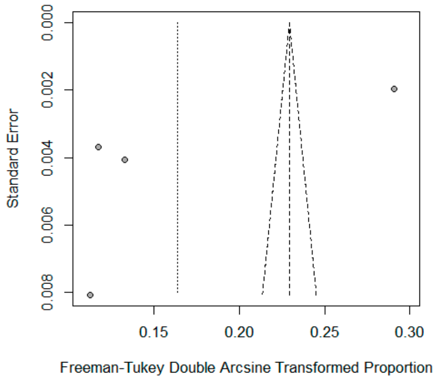

Supplementary Figure S2. Forest plots and funnel plots of Table 3

	Forest plots	Funnel Plots
Overall	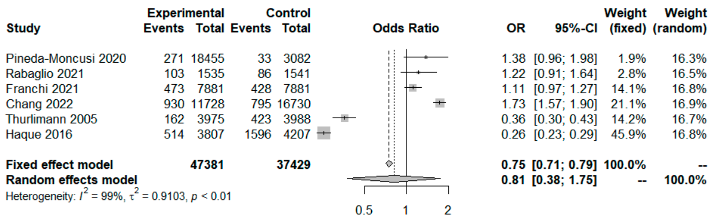	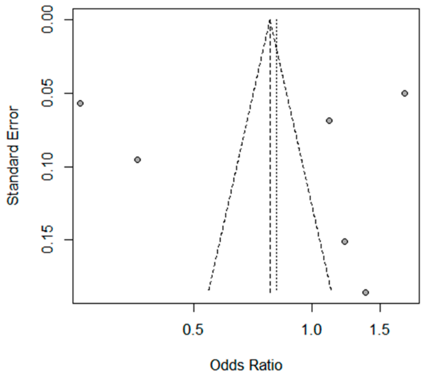
Angina	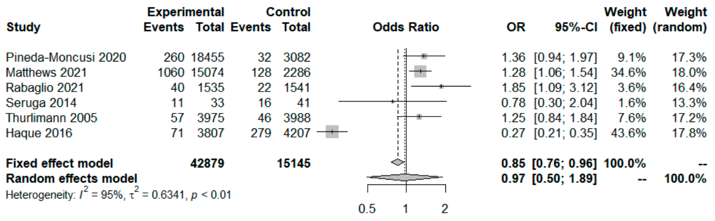	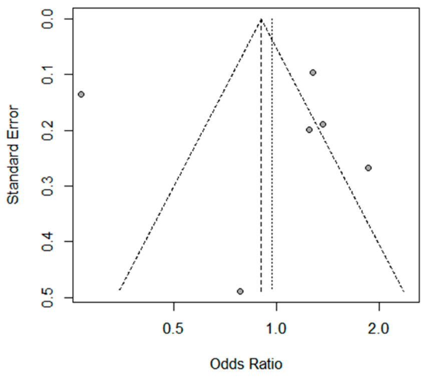
MI	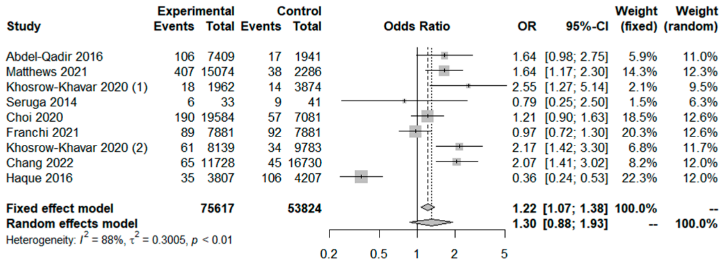	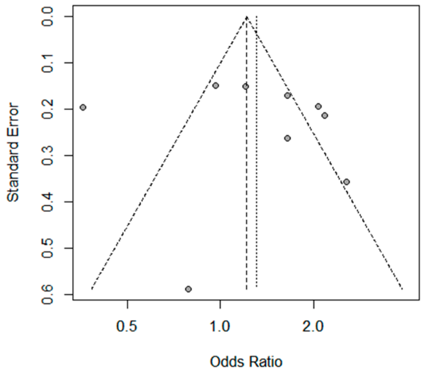

Heart failure

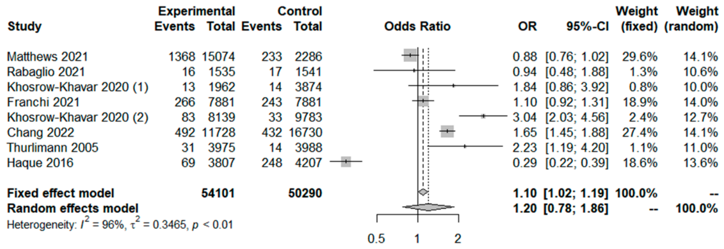

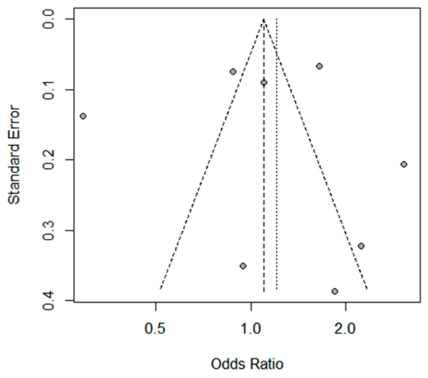

All stroke

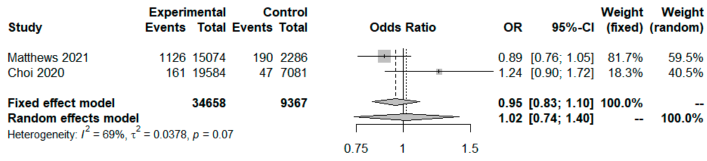

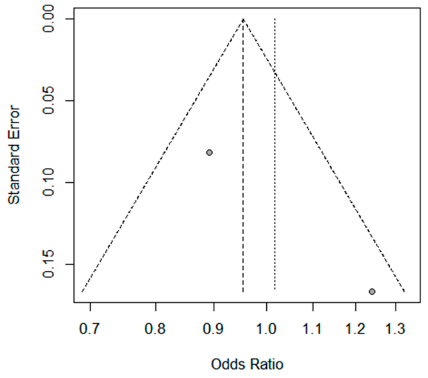

Ischemic stroke

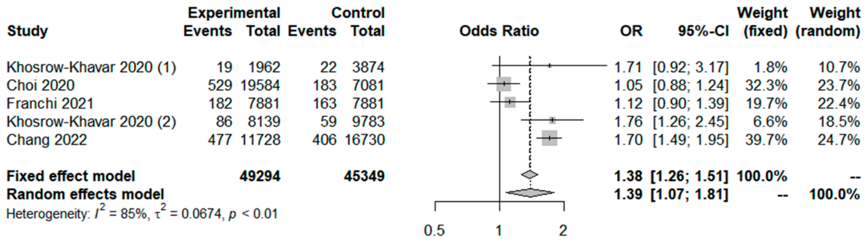

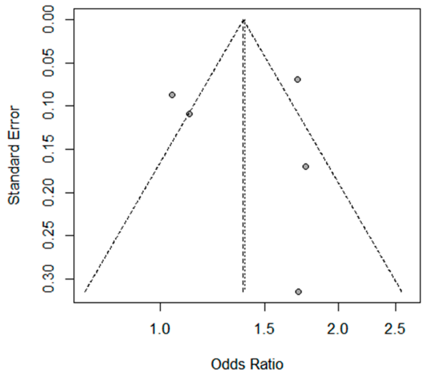

Overall,

VTE

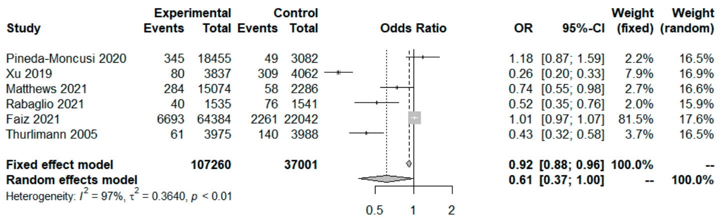

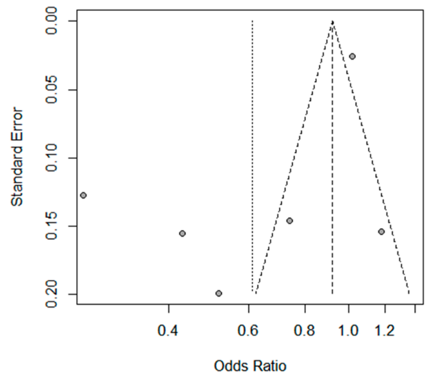

PTE

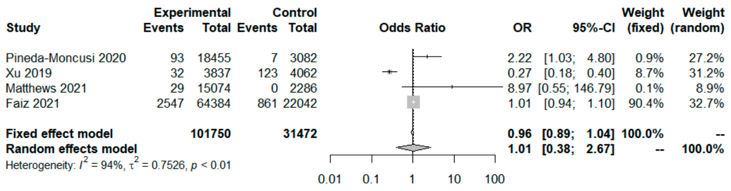

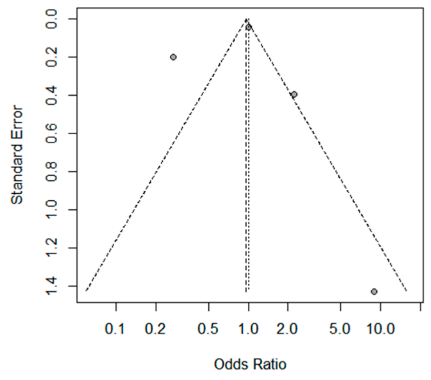

DVT

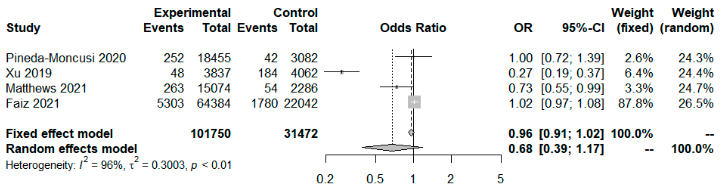

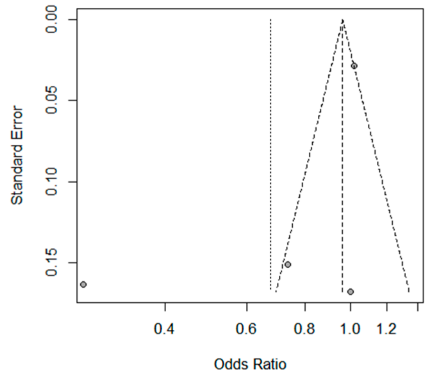

Supplementary Figure S3. Forest plots and funnel plots of Table 4

	Forest plots	Funnel Plots
Comparison with baseline		

HDL 6-mo

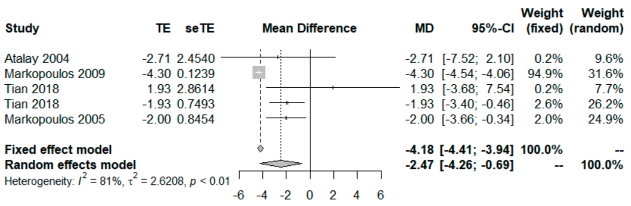

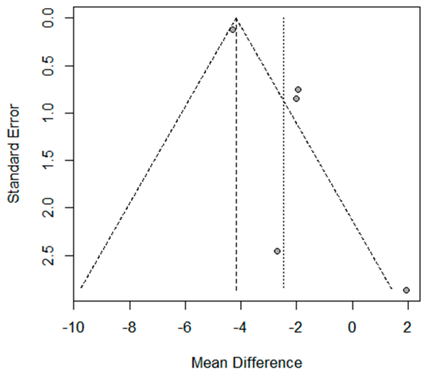

HDL 12-mo

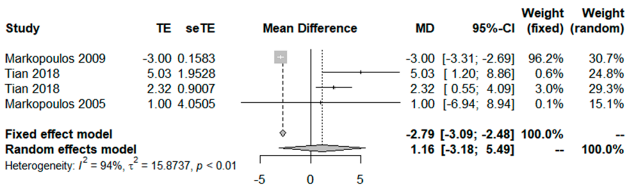

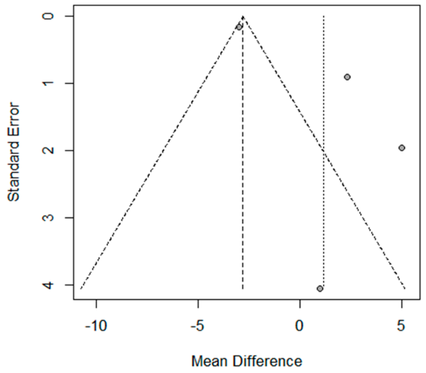

LDL 6-mo

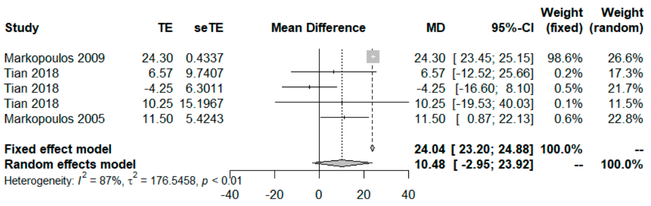

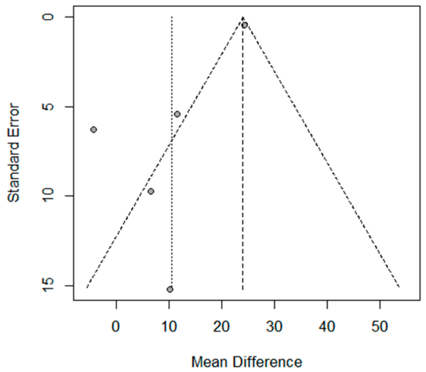

LDL 12-mo

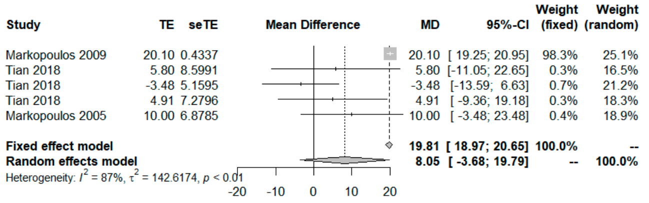

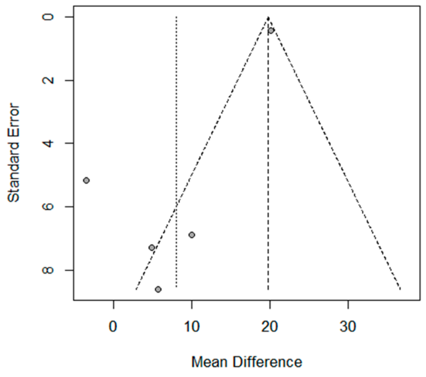

TG 6-mo

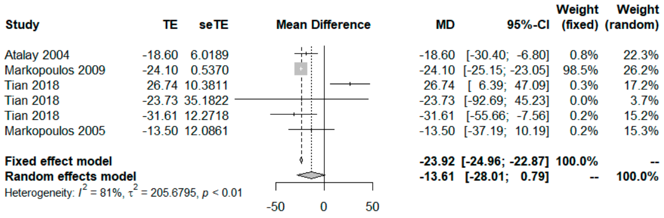

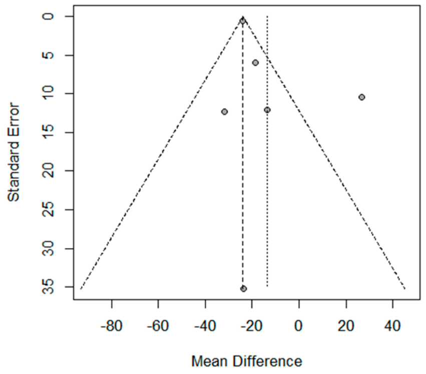

TG 12-mo

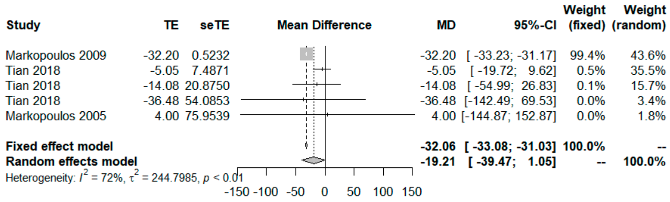

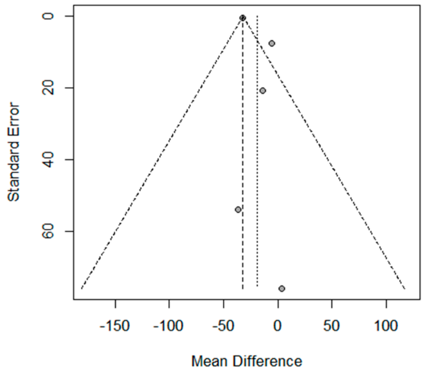

T. chol 6-mo

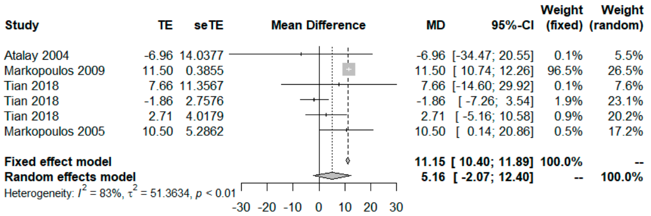

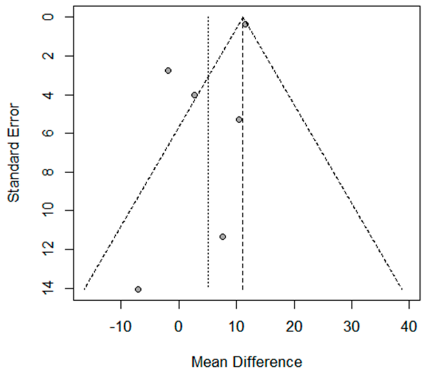

T. chol 12-mo

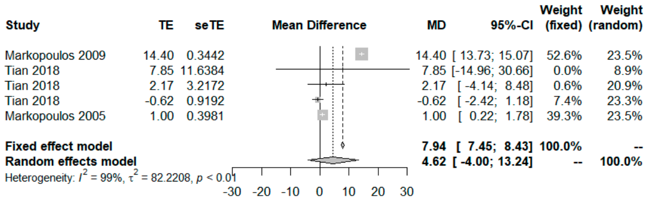

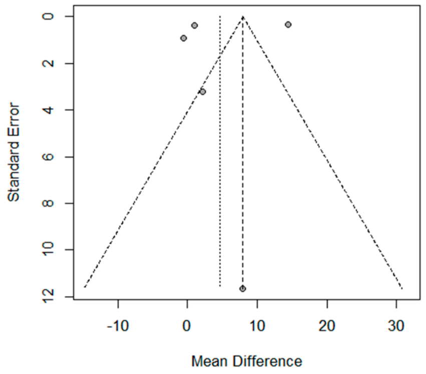

Comparison with tamoxifen

HDL 6-mo

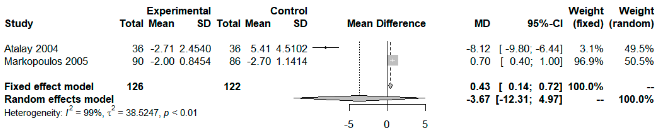

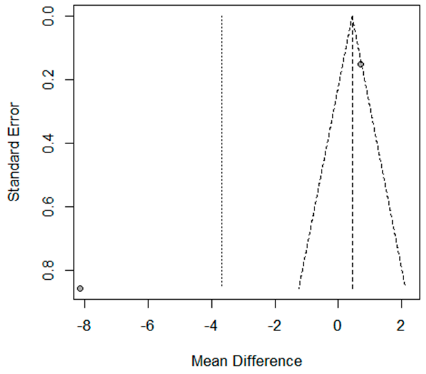

HDL 12-mo

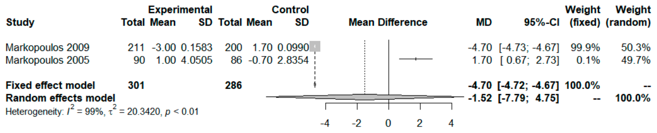

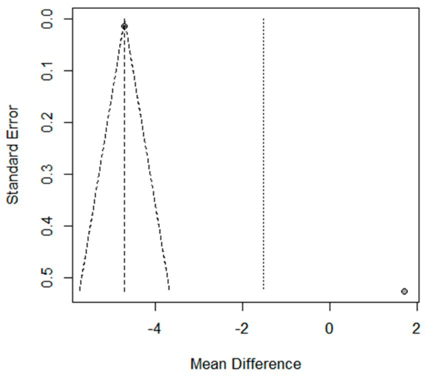

LDL 6-mo

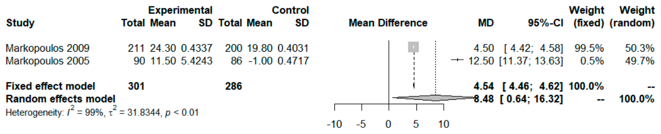

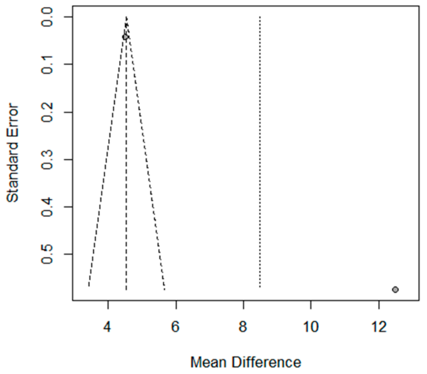

LDL 12-mo

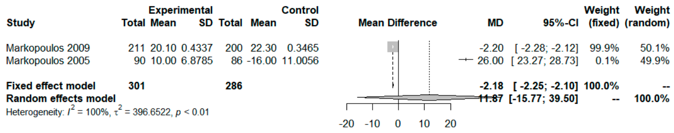

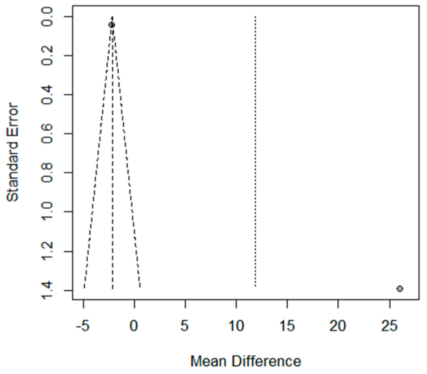

TG 6-mo

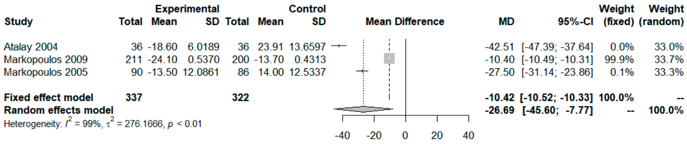

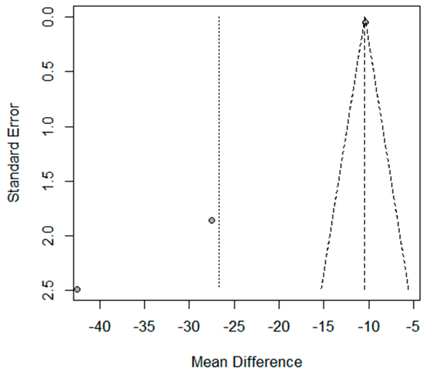

TG 12-mo

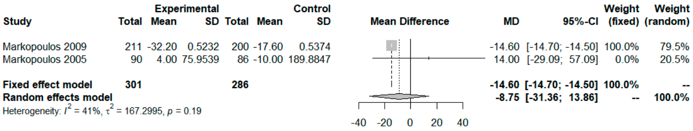

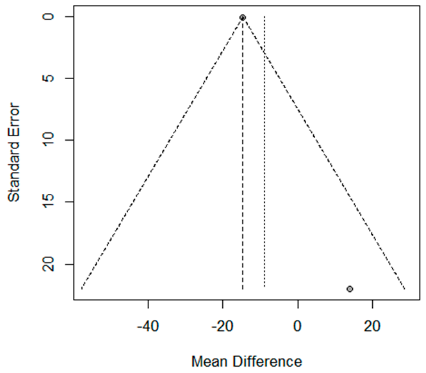

T. chol 6-mo

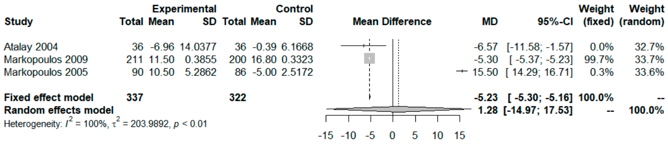

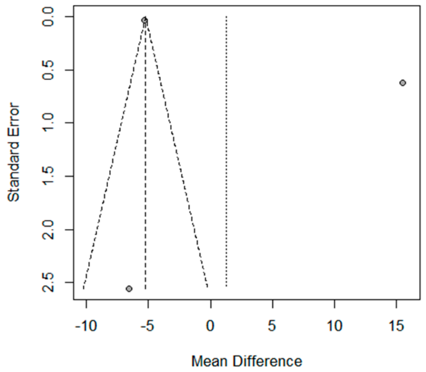

<table>
<tr>
<td>T. chol 12-mo</td>
<td>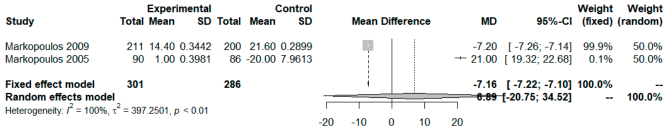
</td>
<td>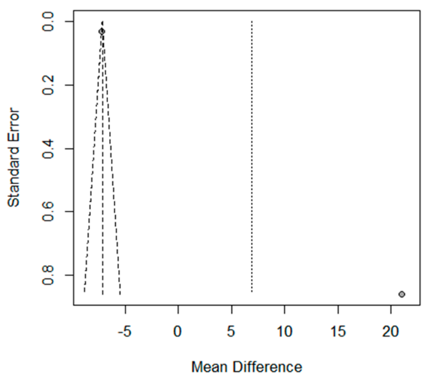
</td>
</tr>
</table>
